# Residue 49 of AtMinD1 Plays a Key Role in the Guidance of Chloroplast Division by Regulating the ARC6-AtMinD1 Interaction

**DOI:** 10.3389/fpls.2021.752790

**Published:** 2021-11-22

**Authors:** Yanhua Zhang, Xiaochen Zhang, Huanshuo Cui, Xinzhu Ma, Guipeng Hu, Jing Wei, Yikun He, Yong Hu

**Affiliations:** College of Life Science, Capital Normal University, Beijing, China

**Keywords:** NCTS motif, punctate structure, AtMinD1, ARC6, chloroplast division, Min complex

## Abstract

Chloroplasts evolved from a free-living cyanobacterium through endosymbiosis. Similar to bacterial cell division, chloroplasts replicate by binary fission, which is controlled by the Minicell (Min) system through confining FtsZ ring formation at the mid-chloroplast division site. MinD, one of the most important members of the Min system, regulates the placement of the division site in plants and works cooperatively with MinE, ARC3, and MCD1. The loss of MinD function results in the asymmetric division of chloroplasts. In this study, we isolated one large dumbbell-shaped and asymmetric division chloroplast Arabidopsis mutant Chloroplast Division Mutant 75 (*cdm75*) that contains a missense mutation, changing the arginine at residue 49 to a histidine (R49H), and this mutant point is located in the N-terminal Conserved Terrestrial Sequence (NCTS) motif of AtMinD1, which is only typically found in terrestrial plants. This study provides sufficient evidence to prove that residues 1–49 of AtMinD1 are transferred into the chloroplast, and that the R49H mutation does not affect the function of the AtMinD1 chloroplast transit peptide. Subsequently, we showed that the point mutation of R49H could remove the punctate structure caused by residues 1–62 of the AtMinD1 sequence in the chloroplast, suggesting that the arginine in residue 49 (Arg49) is essential for localizing the punctate structure of AtMinD1_1__–__62_ on the chloroplast envelope. Unexpectedly, we found that AtMinD1 could interact directly with ARC6, and that the R49H mutation could prevent not only the previously observed interaction between AtMinD1 and MCD1 but also the interaction between AtMinD1 and ARC6. Thus, we believe that these results show that the AtMinD1 NCTS motif is required for their protein interaction. Collectively, our results show that AtMinD1 can guide the placement of the division site to the mid chloroplast through its direct interaction with ARC6 and reveal the important role of AtMinD1 in regulating the AtMinD1-ARC6 interaction.

## Introduction

Chloroplasts, photosynthetic organelles in plants, evolved from an ancestral cyanobacterium harboring their own genomes and internal membrane systems related to the components of modern cyanobacteria (Gray, 1999). During evolution, the majority of prokaryotic genes was lost or transferred from chloroplasts to the eukaryotic host nuclei, enabling adaptation to eukaryotic gene expression systems and gaining cellular functions by targeting their gene products to chloroplasts or other subcellular compartments (Martin et al., 2002). However, in addition to cyanobacterial-derived proteins, a number of non-cyanobacterial-like proteins are targeted to chloroplasts (Martin et al., 2002), implying that modern chloroplasts are maintained by processes that require both inherent prokaryotic systems and acquired eukaryotic systems (Gao et al., 2003; Hashimoto, 2003; Miyagishima et al., 2003; Chen C. et al., 2018; Lee and Hwang, 2018).

Prokaryotes such as bacteria propagate by binary fission. Bacterial cell division is driven by a Z-ring in which the cytoskeletal protein FtsZ, a prokaryotic tubulin homolog, localizes to a midcell and recruits other proteins, forming a divisome (Bi et al., 1991; de Boer, 2010). The assembly of the FtsZ ring is restricted to the midcell by the Min system, comprising MinC, MinD, and MinE, which controls Z-ring positioning by preventing the self-assembly of Z-rings everywhere but at the division site (de Boer et al., 1989; Miyagishima et al., 2005; Lutkenhaus, 2007; Rowlett and Margolin, 2013; Monahan et al., 2014). The cytosolic protein MinC is a direct inhibitor of FtsZ assembly (Hu et al., 1999; Dajkovic et al., 2008) and is activated and recruited to the membrane through direct interaction with the membrane-associated protein MinD (Hu and Lutkenhaus, 2000; Lutkenhaus and Sundaramoorthy, 2003). In *Escherichia coli*, the oscillation of the MinCD complex, driven by the topological factor MinE through direct interaction between MinE and MinD, which causes its time-averaged concentration in the membrane to be highest at cell poles and lowest at the cell center, guides Z-ring formation in the midcell position (Bisicchia et al., 2013).

Chloroplasts, like their cyanobacterial ancestors, propagate by the division of preexisting organelles *via* a binary fission mechanism, and the Z-ring is the first structure assembled at the division site (Miyagishima et al., 2001). In chloroplasts, the Z-ring is formed by the association of FtsZ1 and FtsZ2 heteropolymers on the stromal side of the inner envelope at the division plane *via* the membrane tethering protein ARC6, a bitopic inner transmembrane protein that stabilizes the plastid FtsZ ring and coordinates the internal and external division machineries (Vitha et al., 2003). There are currently two known stroma-division proteins reported to interact with ARC6: FtsZ2 (Lohse et al., 2006) and MCD1 (Chen L. et al., 2018).

Homologs of cyanobacterial MinD have been retained in the green lineage and localize to the stroma, where they play roles in the spatial regulation of chloroplast division and Z-ring placement analogous to those in bacteria (Colletti et al., 2000; Itoh et al., 2001; Vitha et al., 2003; Fujiwara et al., 2004, 2008; Aldridge and Møller, 2005; Glynn et al., 2007). The positioning of AtMinD1, a MinD homolog in Arabidopsis, results in a punctate structure localized to the mid chloroplast and in puncta dispersed on the chloroplast envelope (Fujiwara et al., 2009; Nakanishi et al., 2009b). In the Arabidopsis *arc11* mutant, an *AtMinD1* loss-of-function mutant, chloroplast division is restricted and the division site is displaced, leading to an enlarged, dumbbell-shaped chloroplast phenotype (Fujiwara et al., 2004, 2008). The overexpression or knockdown of AtMinD1 results in fewer and bigger chloroplasts and abnormal FtsZ ring assembly (Colletti et al., 2000; Kanamaru et al., 2000; Dinkins et al., 2001; Vitha et al., 2003). Therefore, the regulation of AtMinD1 protein expression is very important for chloroplast division. The MinE homolog AtMinE1 was identified in Arabidopsis (Itoh et al., 2001; Maple et al., 2002) and confirmed to play a role in the determination of the chloroplast division site, as AtMinE1 overexpression leads to the loss of the topological specificity of MinE (Maple et al., 2002). However, the specific mechanism of action of AtMinE1 is still unknown.

Unlike MinD and MinE, MinC is not a well-conserved protein. During the course of chloroplast evolution, the MinC protein was lost in many land plants. The ARC3 protein has been postulated as a functional replacement for bacterial MinC in land plants (Shimada et al., 2004; Maple et al., 2007) and functions as an FtsZ1 assembly inhibitor (TerBush and Osteryoung, 2012; Zhang et al., 2013; Chen et al., 2019). In bacteria, MinC can only interact with MinD, whereas ARC3 can interact with AtMinD1, AtMinE1, and FtsZ in Arabidopsis (Glynn et al., 2007; Maple et al., 2007). In addition, unlike bacterial MinC, ARC3 co-localizes at the division site and poles of chloroplasts *in vivo* with AtMinD1 and FtsZ (Maple et al., 2007; Zhang et al., 2013). Recently, it was shown that ARC3, *via* the MORN domain, is recruited to the middle of the plastid by the inner envelope membrane protein PARALOG OF ARC6 (PARC6) (Chen et al., 2019). In Arabidopsis, enlarged chloroplast phenotypes that resulted from AtMinD1 overexpression or AtMinE1 deficiency (Colletti et al., 2000; Glynn et al., 2007) could be completely suppressed in the absence of ARC3, suggesting that these proteins function as ARC3 regulators (Zhang et al., 2013).

In addition, the land plant Multiple Chloroplast Division Site 1 (MCD1) is a novel eukaryotic-derived component of the Min system that recruits MinD to the membrane (Nakanishi et al., 2009a) and regulates Z-ring positioning *via* direct interaction with ARC6 in the stroma (Chen L. et al., 2018). Previously, it has been hypothesized that AtMinD1 was recruited to the complexes by interacting with MCD1 after the formation of the ARC6-MCD1 complex, because AtMinD1 can interact with MCD1, and AtMinD1 immunofluorescence cannot be detected in *mcd1*, a MCD1 loss-of-function mutant (Nakanishi et al., 2009b; Chen L. et al., 2018). The accumulation of the MCD1 protein is decreased in *arc11* but increased in AtMinD1-overexpressing mutants, indicating that AtMinD1 can directly affect the protein expression of MCD1 (Nakanishi et al., 2009b). The overexpression of AtMinD1 results in approximately one to two chloroplasts per cell in the wild-type background (Fujiwara et al., 2004; Nakanishi et al., 2009b), and three to five chloroplasts per cell in the *mcd1-1* background (Nakanishi et al., 2009b), suggesting that AtMinD1 may regulate the division of chloroplasts *via* another unknown pathway in the absence of MCD1.

Meanwhile, *arc6* mutants (Pyke et al., 1994) possess one or two giant chloroplasts with fragmented FtsZ filaments; in contrast, ARC6-overexpressing plants, although still bearing enlarged chloroplasts, exhibit exceptionally long FtsZ filaments (Pyke et al., 1994; Vitha et al., 2003). However, AtMinD1 is the exact opposite: in *AtMinD1*-overexpressing plants, as there are one or two giant chloroplasts and fragmented FtsZ filaments, just like in the *arc6* mutant. As the *arc11* mutant gains a polycyclic Z-ring phenotype, we believe that it can be assumed that AtMinD1 and ARC6 act in opposing directions, whereby FtsZ filament formation in the chloroplast is promoted by ARC6 and inhibited by AtMinD1, although there is no strong evidence to uphold this assumption (Vitha et al., 2003). Furthermore, the relationship between ARC6 and AtMinD1 remains unconfirmed.

In this research, we have analyzed the phenotype of *cdm75* and identified its mutation point, which was located in the N-terminal conserved terrestrial specific (NCTS) motif of AtMinD1. Through bioinformatic analysis, it was shown that the NCTS motif is only present in land plants. Moreover, this study revealed the role of residue 49 located in the NCTS motif, a functional domain of AtMinD1, and the effect of this mutation on the interaction of AtMinD1 with other division factors. Furthermore, our findings provide evidence that the connection and close relationship between AtMinD1 and ARC6 are shown to be a vital prerequisite for the process of chloroplast division. The results of this study have demonstrated the core role of residue 49 in the interactions with the chloroplast division-related proteins, ARC6 and MCD1. Collectively, the above findings provide insights into the molecular regulation mechanism guiding the location of the chloroplast division site and reveal that AtMinD1 can enhance the accuracy of plastid division regulation through its interaction with other proteins.

## Materials and Methods

### Plant Materials and Growth Conditions

In this research, *Arabidopsis thaliana Columbia-0* (Col-0) and *Landsberg erecta* (Ler) were used as the wild-type plants, and all of the mutants and transgenic plants used in this study were in the Col-0 background. All the plants were grown in a controlled growth chamber at 21°C under cool white fluorescent light (80–100 μmol m^–2^ s^–1^) under a long day photoperiod (16-h light/8-h darkness).

Arabidopsis seeds, surface-sterilized with 70% (v/v) ethanol and 10% (v/v) sodium hypochlorite, were germinated on half-strength Murashige and Skoog (1/2 MS) medium supplemented with 1% (w/v) sucrose and 0.8% (w/v) agar. The arabidopsis seeds were also grown in garden soil (Basic substrate No. 1; Pindstrup Mosebrug A/S, Denmark) without additional fertilizer, and were maintained in a greenhouse under standard growing conditions (Weigel and Glazebrook, 2010).

### Genetic Analysis of *cdm75* by Mapping

Mutants were isolated from an ethyl methane sulfonate (EMS)-mutagenized Arabidopsis population in the Col-0 background, and the *cdm75* mutant was backcrossed with Col-0 for three generations, and then crossed with the Ler wild-type to obtain F1 seeds. Approximately 2, 100 F2 plants were identified by microscopy and genotyping, and used for the mapping of *cdm75*. The point mutation of *cdm75* was matched in Chr-V using the primers 5-85L/5-85R, nga249L/nga249R, SO262L/SO262R, and 5-58L/5-58R. According to the prediction of examined chloroplast division-related genes, the accurate point mutation of *cdm75* was identified by DNA sequencing.

### Phenotype Analysis

The WT and *cdm75* cells were collected from the second true leaves of 7-day-old seedlings. Leaf tips were fixed in 3.5% glutaraldehyde for 1 h in the dark at room temperature (RT), and then incubated in a 1-M Na_2_EDTA (pH 9) solution for a further 2 h in the dark at RT (Pyke and Leech, 1992). Then, the leaf cell samples were photographed using a microscope. To quantify the chloroplast division phenotype, mesophyll cell plan areas were measured from microscopy images using an image analysis system (Image Analysis System 10.0^1^), and the number of chloroplasts in each cell was counted manually. Thirty mesophyll cells from the leaves of 40-day-old Arabidopsis plants were used for the quantification.

### Sequence Databases, Alignment, and Phylogeny

Protein BLAST (BLASTp) was used to search for homologs of the AtMinD1 protein in the complete sequenced genomes of plants from various biological databases (such as GenBank). Sequences from several species were aligned using ClustalW, and a phylogenetic tree was constructed using the BioNJ software (Gascuel, 1997) by maximum likelihood (ML) analysis.

### Complementation Analysis

The full-length coding sequences of AtMinD1 were amplified using the primer pair W-001/W-002. The PCR product is fused into the *Nco*I/*Bst*EII site of pCAMBIA1302 through digestion. *pCAMBIA1302-AtMinD1* was employed for Agrobacterium-mediated Arabidopsis transformation with the floral dip method (Clough and Bent, 1998; Itoh et al., 2001). In total, 32 transformed (T1) seedlings were selected on MS plates containing hygromycin-B (25 mg/L; Roche, Germany), and T2 to T4 progenies were used for microscopic characterization. Stable T4 seedlings, grown on antibiotic-free MS plates, were subjected to quantitative RT-PCR analysis using the primer pair HY-053/HY-054.

### Isolation of RNA, cDNA Synthesis, and Quantitative Real-Time PCR

Total RNA was isolated using Trizol reagent (Invitrogen, Gaithersburg, MD, United States) in accordance with the instructions of the manufacturer. PrimeScript^TM^ RT Master Mix (TaKaRa Bio Inc., Japan) was used for RNA purification and reverse transcription in accordance with the instructions of the manufacturer. Real-time quantitative reverse transcription-PCR (RT-PCR) was performed using QuantStudio^TM^ 6 Flex Real-Time PCR System (Applied Biosystems, Warrington, United Kingdom) with SYBR Pre-mix Ex TaqTM (TaKaRa Bio Inc., China) in accordance with the instructions of the manufacturer. Relative expression levels of AtMinD1 are presented as values relative to corresponding control samples at indicated times or under the indicated conditions after normalization to actin II transcript levels. The primer pair HY-053/HY-054 was used to detect total *AtMinD1* transcript levels, and HY-055/HY-056 was used to detect endogenous *AtMinD1* transcript levels.

### Immunoblotting Analysis

Immunoblotting analysis of Arabidopsis tissue extracts was performed as described previously (Stokes et al., 2000; Vitha et al., 2001). For the immunoblotting analysis, the total protein was extracted from 1 mg (fresh weight) of tissue. To be more specific, 1 ml of an extraction buffer was added after the sample was ground in liquid nitrogen. After centrifugation at 100,000 *g* for 30 min at 4°C, protein quantification in the extracts was performed using an *RC DC* Protein Assay kit (Bio-Rad, United States). In each test, control samples were loaded to achieve a uniform 80 ng of total protein per lane for immunoblotting with anti-GFP (AE012: AB Clonal, China) and anti-tubulin (T6199; Sigma, United States).

### Yeast Two-Hybrid Assay

The *Saccharomyces cerevisiae* strain Y2H Gold and plasmids pGADT7 and pGBKT7 encoding the Gal4 activation domain and the Gal4 DNA binding domain, respectively, were derived from MATCHMAKER Two-Hybrid System ver. 3 (Clontech Laboratories, United States). Using the primer pair HY-075/HY-076, full-length coding sequences of AtMinD1 and AtMinD1(R49H) were amplified. These PCR products were digested with *Nde*I/*Bam*HI and introduced into the pGBKT7 or pGADT7 vectors, respectively. The full-length coding sequence of ARC6 was amplified using the primer pair Z-001/Z-002, and after digestion of the products and vector by *Nde*I/*Sma*I, alignments were inserted into the corresponding site of pGADT7. Similarly, full-length coding sequences of AtMinE1 and MCD1 were amplified using the primer pairs Z-003/Z-004 and Z-005/Z-006. After digestion of these products and vector by *Nde*I/*Sma*I and *Nde*I/*Bam*HI separately, the alignments were inserted into the corresponding site of pGBKT7, respectively. The yeast strain Y2H Gold was co-transformed with pGADT7- and pGBKT7- derived constructs by PEG/LiAc. Simultaneously, pGADT7 and pGBKT7 empty vectors were also co-transformed as a negative control. Transformants were selected on yeast dropout (synthetic defined, SD) media plates lacking leucine (Leu) and tryptophan (Trp) (Clontech, Japan), and fresh colonies were then dropped onto SD plates without Leu, Trp, and histidine (His) in the presence or absence of Aureobasidin A (AbA). The cell plates were cultured for 3–5 days at 28°C.

### Pull-Down Assay

To express recombinant His-ARC6, His-MCD1, and His-AtMinE1 in *Escherichia coli*, full-length coding sequences of ARC6, MCD1, and AtMinE1 were amplified using the corresponding primer pairs Z-001/Z-009, Z-003/Z-010, and Z-005/Z-006. After the digestion of these PCR products with *Nde*I/*Sal*I, *Nde*I/*Sal*I, and *Nde*I/*Bam*HI, respectively, these segments were inserted into the corresponding site of pET28a (+)separately. Similar to the construct GST-AtMinD1, the full-length coding sequences of AtMinD1 was amplified using primer Z-007/Z-008, and then the PCR product was introduced into the *Bam*HI/EcoRI digested pGEX-KG. Moreover, all of these products reconstructed with the vector and pGEX-KG empty vectors were expressed in Transetta (DE3) *E. coli* (TransGen, China).

*Escherichia coli* (OD_600_ = 0.7), expressing HisARC6, His-MCD1, and His-AtMinE1, was treated with 0.6 mM IPTG, and GST-AtMinD1 was treated with 1 mM IPTG, followed by incubation for 14–16 h at 16°C. Then, the total protein was extracted from the cells by sonication in a lysis buffer (140 mM NaCl, 2.7 mM KCl, 10 mM Na_2_HPO_4_, 1.8 mM KH_2_PO_4_, and 1 mM PMSF). After centrifugation at 100,000 *g* for 30 min at 4°C, the supernatant was collected and used in the GST pulldown assays. In the assay, 50-μl samples of 50% slurry of Glutathione Sepharose 4B beads (GE Healthcare, United States) were equilibrated in the lysis buffer. After mixing 1 ml of crude cell extract supernatants with GST or GST-AtMinD1 fusion proteins, the slurry samples were incubated for 4 h at 4°C on a nutator. After four washes in 1 ml of a wash buffer (300 mM NaCl, 2.7 mM KCl, 10 mM Na_2_HPO_4_, 1.8 mM KH_2_PO_4_, and 1 mM PMSF, 0.1% NP-40), the beads were mixed with 1 ml of total protein extracts containing the His-ARC6, His-MCD1, or His-AtMinE1 fusion protein in a final volume of 1 ml in the lysis buffer for 4 h at 4°C. Subsequently, the beads were washed three times with 1 ml of the wash buffer and then mixed with a 2 × SDS loading buffer. The proteins were resolved on 10% SDS-PAGE gels and subjected to immunoblotting analysis with antibodies specific for the His tag (Sigma; H1029) and GST tag (HX1807; Huaxingbio, China).

### Transient Expression Analysis of Arabidopsis Protoplasts

To test the localization of different fragments of AtMinD1, different AtMinD1 fragment sequences, comprising the residues 1–49, 1–49(R49H), 1–61, 1–61(R49H), 1–62, 1–62(R49H), 1–64, 1–64(R49H), were amplified with the gene-specific primers W-001/M-011, W-001/M-012, W-001/M-015, and W-001/M-016, and fused with the full-length coding region of GFP. After digestion with *Nco*I/*Eco*RI, these fragments were inserted into pSAT1-GFP, which is a reconstructed vector, by replacing the cGFP^C^ fragment of pSAT1-cGFP^C^ with GFP controlled by the 35S promoter. Accordingly, these gene reconstructions generated pSAT1-AtMinD1_fragment_-GFP.

For the bimolecular fluorescence complementation (BiFC) experiment, the fragments of AtMinD1_1__–__62_ and the full-length coding sequences of ARC6, MCD1, and AtMinE1 were amplified using the primers pairs M-021/M-022, M-024/M-025, M-026/M-027, and M-028/M-029, respectively. After digestion with *Nco*I/*Sal*I, the amplified products were cloned into pSAT1-cGFP^C^ and/or pSAT1-cGFP^N^ to reconstruct the transgenic vectors (Maple et al., 2005; Xu et al., 2005), such as pSAT1-cGFP^C^-AtMinD1_1__–__62_, pSAT1-cGFP^C^-*cdm75*_1__–__62_, pSAT1-cGFP^N^-ARC6, pSAT1-cGFP^N^-AtMinE1, and pSAT1-cGFP^N^-MCD1. All the constructs were verified by sequencing.

As previously described (Yoo et al., 2007), the protoplasts, isolated from the leaves of 4-week-old Arabidopsis plants in the Col-0 background were counted with a hemocytometer and diluted to 2.5 × 10^5^ protoplasts ml^–1^. For each transformation, 200 ml of protoplasts were transformed with a total of 20 μg plasmid and then incubated in the dark prior to examination. Based on the established methodology, the reconstructed constructs and empty vector (the negative control) were transfected into Arabidopsis protoplasts. The cells were then examined using a laser scanning confocal microscope (Zeiss LSM 780; Zeiss, Germany). Emission wavelengths between 503 and 518 nm were used to detect GFP, and wavelengths between 590 and 608 nm were used to detect chlorophyll auto-fluorescence. The obtained images were subsequently analyzed using the Adobe Photoshop CS5.1 software.

### Databases and Software Tools

The DNA and protein sequence databases of the National Center for Biotechnology Information^2^ were accessed. Preliminary sequence data for most cyanobacterial genomes were obtained from the Department of Energy Joint Genome Institute^3^ and from the Kazusa DNA Research Institute of Japan^4^. The *Chlamydomonas reinhardtii* genomic sequence was accessed at http://www.biology.duke.edu/chlamy_genome/blast/blast_form.html. Protein and DNA sequence similarity searches were performed using Basic Local Alignment Search Tool (TBLASTN and BLASTN; Altschul and Lipman, 1990). To predict subcellular protein targeting, ChloroP version 1.01 (Emanuelsson et al., 1999), TargetP V2.0 (Almagro Armenteros et al., 2019), and Predotar version v.1.04^5^ were used. The prediction of transmembrane domains in the protein sequences was performed with HMMTOP version 2.0 (Tusnády and Simon, 2001), TMHMM version 2.0 (Krogh et al., 2001), DAS (Cserzo et al., 1997), SOSUI (Hirokawa et al., 1998), Split (Juretic et al., 2002), TMPRED^6^, and TopPred2 (Claros and von Heijne, 1994).

The identification of conserved domains is facilitated by searches in the Pfam^7^ and Predict Protein^8^ databases (Rost et al., 2003). Sequence manipulation, multiple alignment, and shading of aligned sequences were performed using BioEdit 5.09.

## Results

### Dividing Chloroplasts in *cdm75* Show Dumbbell-Shaped and Enlarged Morphologies, and *cdm75* Is an *AtMinD1*-Related Mutant

**FIGURE 1:**
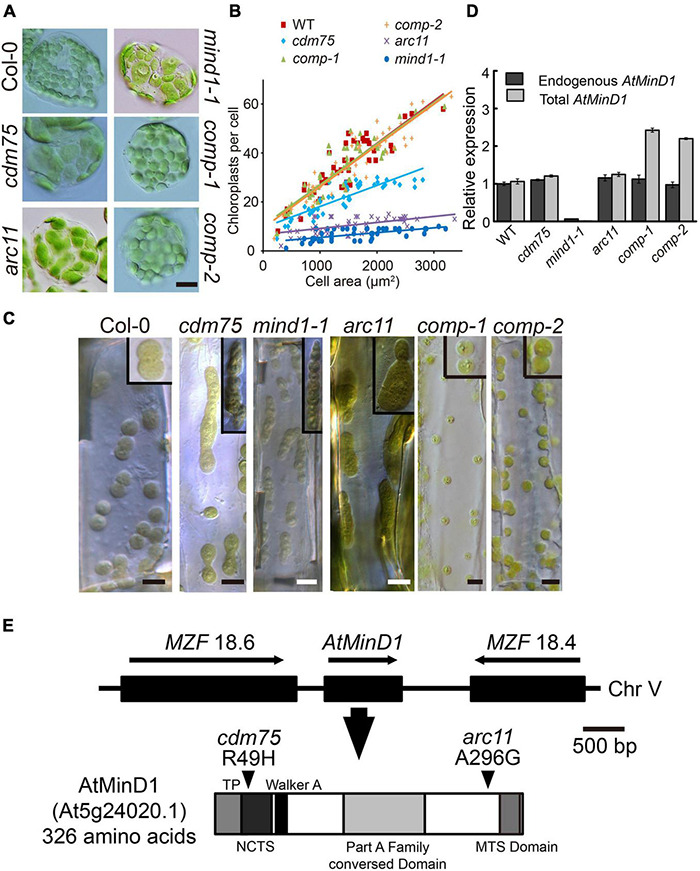
Analyses of phenotype and mutation point of AtMinD1 in *cdm75*. **(A)** Different chloroplast division phenotypes of Arabidopsis leaf mesophyll cells in 25-day-old plants for *Arabidopsis thaliana Columbia-0* (Col-0) (wild-type), *cdm75*, *arc11*, *mind1-1*, and *cdm75* complemented with wild-type *AtMinD1* transgene plants [35S-AtMinD1-HA/*cdm75*-1 (*comp-1*) and 35S-AtMinD1-HA/*cdm75*-2 (*comp-2*)]. Scale bars = 10 μm. **(B)** Graph of chloroplast number relative to cell size in the 25-day-old plants. Best-fit lines have slopes of 0.0181 (*R^2^* = 0.79), 0.092 (*R^2^* = 0.76), and 0.0173 (*R^2^* = 0.75) for the lines shown in **(A)**. **(C)** Differential interference contrast (DIC)-single optical sections of dividing chloroplasts in Arabidopsis leaf hypocotyls cells of the lines shown in **(A)**. Scale bars = 5 μm. **(D)** Quantification of endogenous and total AtMinD1 transcripts in the Arabidopsis plants. Total RNAs from whole seedlings of Arabidopsis, the lines shown in **(A)**, were analyzed with a real-time PCR system. Primer sets specific to the coding region and the 3’-UTR of *AtMinD1* were employed to monitor total (dark gray bars) and endogenous (light gray bars) *AtMinD1* transcript levels, respectively. Amounts of *AtMinD1* transcripts relative to *AtUBQ* are shown as the mean ± SD (with WT = 1) from three different plant samples. **(E)** Chromosomal location of AtMinD1 and the domain structure of its product. Single base substitution of *AtMinD1* at position arginine (Arg) (49) in the specific fragment, changing Arg to histidine (His), is indicated by arrowheads.

Research on the mechanisms of chloroplast division is well established; hence, it is known that the process of chloroplast division is controlled by many chloroplast-division-related proteins (Chen C. et al., 2018). However, the functional mechanisms of all of these proteins have not been completely identified; one such protein is *AtMinD1* (Vitha et al., 2003; Ishikawa et al., 2019). In this study, we obtained a mutant with abnormal chloroplast division named Chloroplast-Division-related Mutant 75 (*cdm75*), that was found to have a typical ethyl methane sulfonate (EMS)-induced point mutation. For the efficient characterization of division states of *cdm75*, we made use of hypocotyls and leaf cells at an early stage of seedling development. *cdm75* is characterized by enlarged or dumbbell-shaped chloroplasts with constriction defects (Figures 1A,C). After the chloroplasts per cell in WT and *cdm75* mutant leaves were counted, we found that in the fully expanded leaves of 3- to 4-week-old plants, there were, on average, approximately 45 chloroplasts per cell in the WT (Col-0) plants and approximately 17 chloroplasts per cells in the *cdm75* mutant (Figure 1B). These results confirm the abnormal division of chloroplasts in *cdm75*.

Following adequate cleansing of the background of *cdm75* and without removing its correlated phenotype, the mutation site of *cdm75* was detected with a mapping method. The *cdm75* locus was mapped onto chromosome V (Figure 1E), which is in close proximity to the *AtMinD1* locus (Marrison et al., 1999). According to the sequence analysis of *AtMinD1* in *cdm75*, the results revealed that a single nucleotide substitution was present, namely, guanine to adenine at the 146th site of its cDNA sequence, resulting in a missense R49H mutation at the N-terminal of AtMinD1 (Figure 1E). By comparison of the chloroplast division phenotype of *cdm75* with other Arabidopsis *AtMinD1* antisense plants, it was shown that the asymmetric division of *cdm75* mesophyll chloroplasts was similar to that of the previously reported *arc11* and *mind1-1* mutants, although the relative chloroplast size of *cdm75* was smaller (Figures 1A,B; Colletti et al., 2000; Fujiwara et al., 2004). Given that the *mind1-1* mutant was derived from the Wassileskija (WS) ecotype, whereas *cdm75* was derived from the Col-0 background, we used a newly identified allele of *AtMinD1*, in the Col ecotype in our mutant screen, for a better comparison of the phenotypes. Based on the quantification of *AtMinD1* transcripts, the statistics indicated that the transcript abundance in Col-0 and *cdm75* seedlings was almost identical to that in *arc11*, which has a single nucleotide mutation at A296G (Figure 1D).

To test whether the *cdm75* phenotype was indeed caused by the R49H mutation in AtMinD1, a wild-type *AtMinD1* transgene was introduced into the nuclear genome of *cdm75* plants. The chloroplast morphology of transgenic plants showed that partial or full complementation phenotypes could be observed in two independent transgenic lines (*comp-1* and *comp-2*), showing stable complementation phenotypes (Figures 1A,C), whereas more than 18 lines showed an apparent inhibited division phenotype (Supplementary Figure 1). In addition, the quantitative RT-PCR analyses of total and endogenous AtMinD1 transcript levels confirmed that the transgenic plants (*comp-1* and *comp-2*) showing successful complementation had AtMinD1 transgene expression levels similar to those of the wild-type (Colletti et al., 2000; Dinkins et al., 2001). Meanwhile, the total *AtMinD1* relative expression level was twice as high as endogenous *AtMinD1* relative expression (Figure 1D). Expression pattern is one of the key factors needed to recover a phenotype (Fujiwara et al., 2004). Overall, these findings showed that *cdm75* was a loss-of-function mutant of *AtMinD1*, and that the observed phenotype was caused by a single R49H mutant.

### Point Mutation of the *cdm75* Mutant (R49H) Is Localized to the N-Terminal Conserved Terrestrial Specific Motif

Division Mutant 75 is encoded by a known gene of the chloroplast division. However, its chloroplast phenotype is slightly different from that of the other related mutant, *arc11*, as its point mutation is located in the N terminal of AtMinD1 (Figure 1). Furthermore, previous studies have not revealed the function of this N-terminal conserved domain, because it always regarded as a transit peptide that would be cleaved (Kanamaru et al., 2000; Fujiwara et al., 2004). Thus, we are convinced that an analysis of MinD sequences from different species, especially the N-terminal protein sequence of MinD, is definitely important, as the function of AtMinD1 can be lost directly by the point mutation of *cdm75*. Therefore, we considered the alignment of the MinD protein sequence in different MinD species and analyzed the features of this sequence.

**FIGURE 2:**
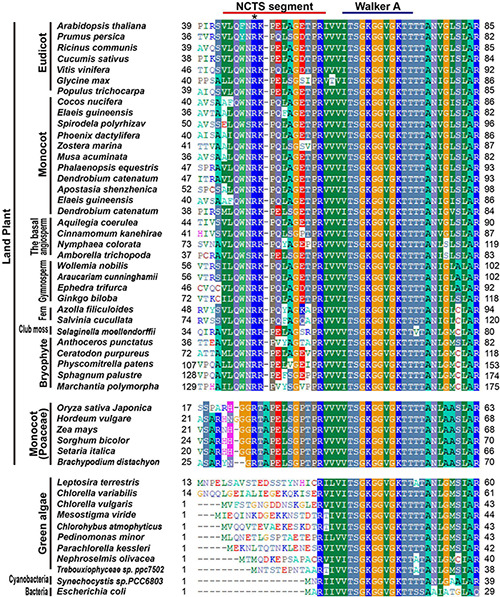
Protein sequence alignment analysis of MinD in different species. An alignment of the extreme N-terminal region of AtMinD1 with the corresponding region of MinD from various organisms is shown. N-terminal Conserved Terrestrial Specific (NCTS) domain (44th–58th) in land plants has been underlined. Asterisk position was the point mutation site of *cdm75*, which is a mutant of AtMinD1.

In the comparison of bacteria, cyanobacteria, green algae, and land plants, an extended alignment of the N-terminal of MinD was found (Figure 2). Interestingly, the new conserved segment, residues 44–58 in the AtMinD1, is only found in terrestrial plants, so we assume it is evolved from the common ancestor of the land plant, and name it as NCTS (N-terminal Conserved Terrestrial Specific) motif (Figure 2). Unusually, although almost all land plants share homology in the N-terminal of the MinD protein, an individual gramineous segment that was non-homologous with other land plants was found in Poaceae plants (Figure 2). Furthermore, to examine the evolution of MinD, we compared AtMinD1 with 84 different MinD proteins from bacteria to plants and divided them into different groups based on their protein alignment (Supplementary Table 1). The resulting unrooted dendrogram clearly showed that the MinD protein of all eukaryotic plants and prokaryotes were initially separated (Supplementary Figure 2). In addition, by splitting the branch of green algae, all the MinD proteins in land plants were classified into a group. Meanwhile, the alignments that contained a gramineous motif in the N-terminal were grouped within a branch; others with an acquired conserved segment that was found in almost all land plants, were closely related (Supplementary Figure 2). Based on the analysis and prediction of protein alignment, the bioinformatic analyses strongly support the hypothesis that the N-terminal of AtMinD1 contains a conserved functional motif, the NCTS motif, which results in the gain of a specific function of chloroplast division.

### Mutation of Residue 49 of AtMinD1 Can Disrupt the Punctate Structure of AtMinD1_1__–__62_ in Arabidopsis Chloroplasts

**FIGURE 3:**
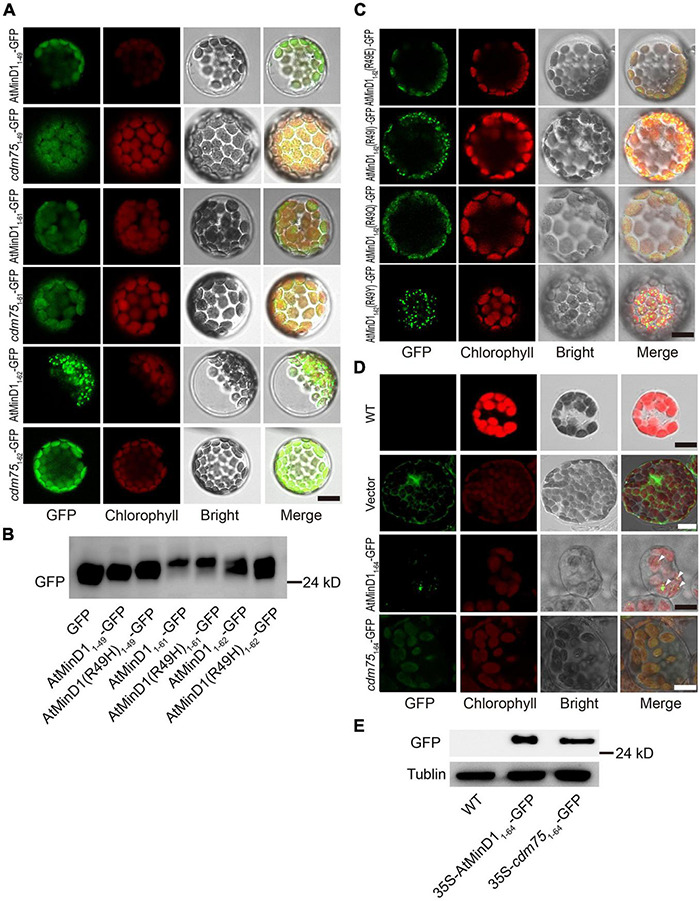
Analyses of the transit peptide and localization of the specific fragment in AtMinD1 with GFP. **(A)** Fluorescence micrographs showing the different lengths of fragments fused with GFP transiently expressed in Arabidopsis protoplasts, which include AtMinD1_1__–__49_-GFP, *cdm75*_1__–__49_-GFP, AtMinD1_1__–__61_-GFP, *cdm75*_1__–__61_-GFP, AtMinD1_1__–__62_-GFP, and *cdm75*_1__–__62_-GFP. Scale bars = 10 μm. **(B)** Total proteins from the transfected protoplasts shown in **(A)** were extracted for further immunoblotting analysis. Bands on the blots were detected using GFP antibody. **(C)** Fluorescence micrographs showing the different mutation of residue 49 in AtMinD1_1__–__62_ and fused with GFP transiently expressed in Arabidopsis protoplasts, which include AtMinD1(R49E)_1__–__62_-GFP, AtMinD1(R49I)_1__–__62_-GFP, AtMinD1(R49Q)_1__–__62_-GFP, and AtMinD1(R49Y)_1__–__62_-GFP. Scale bars = 10 μm. **(D)** Different fluorescent phenotypes of Arabidopsis leaf mesophyll cells, which include wild-type Arabidopsis and over-expressed *35S-AtMinD1*_1__–__64_-*GFP* or *35S-cdm75*_1__–__64_-*GFP* or empty-vector Arabidopsis. Triangle position was the fluorescent dots of *35S-AtMinD1*_1__–__64_-*GFP*. Scale bars = 10 μm. **(E)** Immunoblotting analysis showing the protein of wild-type and over-expressed lines *35S-AtMinD1*_1__–__64_-*GFP* and *35S-cdm75*_1__–__64_-*GFP*, shown in **(D)**, which were extracted from 1 mg (fresh weight) of leaves. Tubulin was used as a loading control.

AtMinD1 is a chloroplast inner-membrane protein encoded by a nuclear gene. Therefore, AtMinD1 is transferred to chloroplast stroma through its transit peptide (TP), a type of peptide with an elongated N-terminal (Maple et al., 2002, 2005; Martin et al., 2002; Vitha et al., 2003; Fujiwara et al., 2004). Previous studies have concluded that the segment comprising residues 1–64 of the N-terminal of AtMinD1 was the TP, as predicted by TargetP and ChloroP (Kanamaru et al., 2000; Fujiwara et al., 2004). However, we predicted the TP of AtMinD1 using TargetP2.0 (Almagro Armenteros et al., 2019) and found that a shorter segment, from residues 1–43 of AtMinD1, was the chloroplast TP, and the cleavage site was between Ser43 and Val44 (Supplementary Table 2). To verify whether a mutation at residue 49 in AtMinD1 would affect the function of the TP, a fusion protein with different lengths of peptides, such as the N-terminal of AtMinD1, linked with GFP was introduced into the Arabidopsis protoplast by transient expression *in vivo*. We found that the fused protein, comprising residues 1–49 of AtMinD1 fused with the GFP protein (AtMinD1_1__–__49_-GFP) could be transited into chloroplasts. Moreover, when the missense mutation site of *cdm75* was located at residue 49 (R49H), the transit function was retained (Figure 3A). Therefore, it is clear that mutation of amino acid 49 does not prevent AtMinD1 from entering the chloroplast.

In Arabidopsis, AtMinD1 is localized to division sites and forms punctate structures dispersed on the chloroplast inner envelope (Nakanishi et al., 2009b). To detect the position of the N-terminal of AtMinD1 in Arabidopsis, different lengths of peptides fused with GFP were introduced into the Arabidopsis protoplast by transient expression *in vivo*. We found if the peptide of the N-terminal AtMinD1 was less than 62 amino acids, such as the initial 61 amino acids, despite whether it was from the wild-type (AtMinD1_1__–__61_-GFP) or the *cdm75*, AtMinD1(R49H) mutant (*cdm75*_1__–__61_-GFP), the fusion protein could still be transited into chloroplast, but the punctate structures could not be found (Figure 3A). Interestingly, an unexpected phenomenon was found, whereby if the peptide from residues 1–62 of the N-terminal of AtMinD1 was fused with GFP (AtMinD1_1__–__62_-GFP), the fusion protein was condensed to punctate structures in the chloroplast (Figure 3A). Conversely, punctate localization can be influenced, as the peptide that is the component of fusion peptide has been replaced by the copy from *cdm75* (*cdm75*_1__–__62_-GFP), which has a point mutation in AtMinD1 (R49H) (Figure 3A). Moreover, when changing the nature of the amino acid, for example, to acidic (glutamic acid, E), polar (glutamine, Q), and non-polar (isoleucine, I) residues, or those with special properties (tyrosine, Y), at residue 49 of the AtMinD1 protein, the punctate phenotype was not always formed. Similar to *cdm75*_1__–__62_-GFP, the punctate phenotype was not formed for some amino acids (E and Q), but was formed for others (I and Y), similar to AtMinD1_1__–__62_-GFP (Figure 3C).

In previous research, although the peptide constructed by the initial 62 amino acids was found to contribute to the transit of AtMinD1 to the chloroplast, the punctate phenotype has not been observed. To analyze the effects of the transient expression, two fusion proteins, AtMinD1_1__–__64_-GFP and *cdm75*_1__–__64_-GFP driven by the constitutively active 35S promoter, were transformed into wild-type (Col-0). A Western blotting analysis showed that the fusion proteins of 35S-AtMinD1_1__–__64_-GFP and 35S-*cdm75*_1__–__64_-GFP were expressed in the plants (Figure 3E). Simultaneously, fluorescence localization was observed, and it was found that the punctate phenotype was also obvious with the constitutive overexpression of 35S-AtMinD1_1__–__64_-GFP; although we could not find any clear fluorescent positions in the 35S-*cdm75*_1__–__64_-GFP transgenic lines (Figure 3D), the same phenomenon has been observed in transient protoplast transformation (Supplementary Figure 3). Therefore, based on the above results, we believe that amino acid 49 does not affect the transit peptide function of AtMinD1, and that it is only related to the punctate localization of the N-terminal of AtMinD1.

### Mutation of Residue 49 in AtMinD1 Can Influence the Interaction Between AtMinD1_1__–__62_ and Chloroplast Division-Related Proteins, Namely, ARC6, MCD1, and AtMinE1, *in vivo*

**FIGURE 4:**
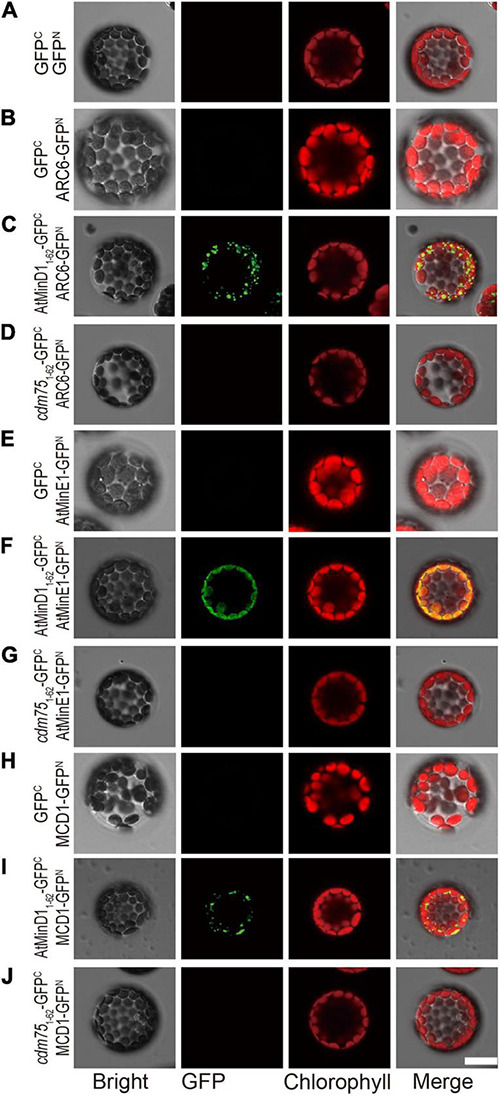
Interaction between AtMinD1_1__–__62_ or *cdm75*_1__–__62_ and chloroplast division-related proteins (ARC6, MCD1, and AtMinE1) determined using the BiFC assay in Arabidopsis protoplasts. Bimolecular fluorescence complementation (BiFC) assays were performed by co-expression of the indicated combinations of plasmids in protoplasts isolated from wild-type Arabidopsis plants. Fluorescence of the reconstituted GFP fluorophore was detected by epifluorescence microscopy. Different types of protoplasts with **(A)** co-expression of 35S-GFP^C^ and 35S-GFP^N^; **(B)** co-expression of 35S-GFP^C^ and 35S-ARC6-GFP^N^; **(C)** co-expression of 35S-AtMinD1_1__–__62_-GFP^C^ and 35S-ARC6-GFP^N^; **(D)** co-expression of 35S-*cdm75*_1__–__62_-GFP^C^ and 35S-ACR6-GFP^N^; **(E)** co-expression of 35S-GFP^C^ and 35S- AtMinE1-GFP^N^; **(F)** co-expression of 35S-AtMinD1_1__–__62_-GFP^C^ and 35S-AtMinE1-GFP^N^; **(G)** co-expression of 35S-*cdm75*_1__–__62_-GFP^C^ and 35S- AtMinE1-GFP^N^; **(H)** co-expression of 35S-GFP^C^ and 35S-MCD1-GFP^N^; **(I)** co-expression of 35S-AtMinD1_1__–__62_-GFP^C^ and 35S-MCD1-GFP^N^; and **(J)** co-expression of 35S-*cdm75*_1__–__62_-GFP^C^ and 35S-MCD1-GFP^N^. GFP and chlorophyll signals were colored green and red, respectively. Scale bars = 10 μm.

To analyze the functional influence of AtMinD1 caused by point mutations, database searching based on protein structure and function predictions (the Pfam database, see text footnote 7 or 8) (Rost et al., 2003) was conducted to analyze the NCTS motif, which includes amino acid 49 of AtMinD1, the site of *cdm75* point mutation. The results show that the residue SVLQ in the NCTS motif (from 35 to 46 of AtMinD1) is a part of α1 helix, and that the other residues of the NCTS motif (from 47 to 58 of AtMinD1) is the disorder coil between α1 and β2 (Supplementary Figure 4). We presumed that the NCTS motif may interact with other proteins to form specific punctate structures (Figure 3A). To confirm the interaction between the peptides AtMinD1_1__–__62_-GFP and *cdm75*_1__–__62_-GFP and chloroplast division-related proteins (AtMinE1, MCD1, and ARC6) *in vivo*, we performed a bimolecular fluorescence complementation (BiFC) assay based the co-expression of the reconstitution of GFP fluorescence when non-fluorescent N-terminal GFP (GFP^N^) and C-terminal GFP (GFP^C^) fragments were brought together by two interacting proteins in living protoplasts. The peptide comprising the initial 62 amino acids of AtMinD1 was fused with the GFP^C^ tag at the C-terminal (AtMinD1_1__–__62_-GFP^C^ and *cdm75*_1__–__62_-GFP^C^), and the chloroplast division-related protein was fused with the GFP^N^ tag at the C-terminal (AtMinD1-GFP^N^, AtMinE1-GFP^N^, MCD1-GFP^N^, and ARC6-GFP^N^). The assay results showed that AtMinD1_1__–__62_ could interact with AtMinE1, MCD1, ARC6, and that the fluorescence caused by the interaction disappeared, indicating that the point mutation of AtMinD1_1__–__62_ (R49H) could impair its interaction with chloroplast division-related proteins (Figure 4). It should be noted that the interaction between AtMinD1_1__–__62_ and ARC6 was extremely condensed to the punctate structure phenotype (Figure 4), which was similar to the indicated localization of this segment (Figure 3A). Overall, our data strongly suggest that this NCTS motif of AtMinD1 can interact with AtMinE1, MCD1, and ARC6.

### Mutation of Residue 49 Reduces the Direct Interaction of AtMinD1 With ARC6 *in vivo* and *in vitro*

**FIGURE 5:**
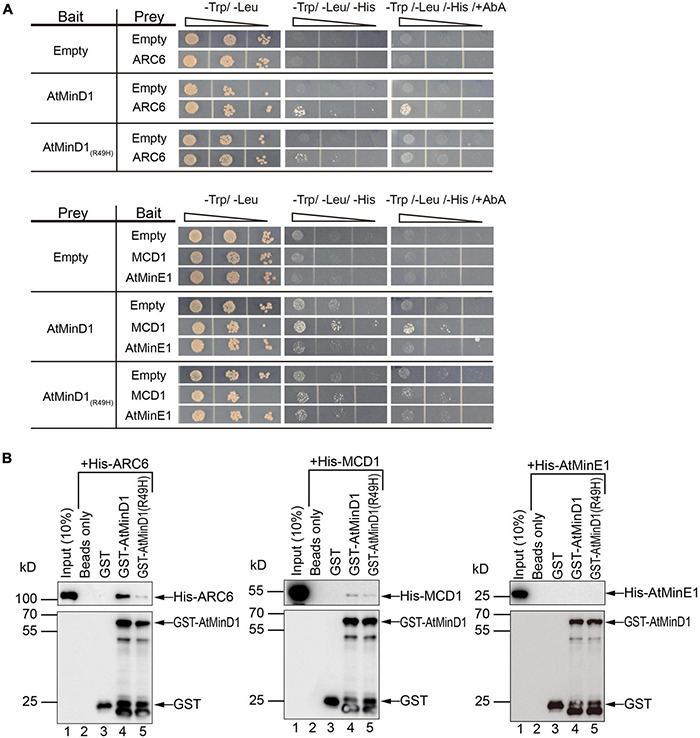
Interaction between AtMinD1 or *cdm75* and chloroplast division-related proteins (ARC6, MCD1, and AtMinE1). **(A)** Yeast two hybrid system investigating the interactions of AtMinD1 or *cdm75* with ARC6, and MCD1, and AtMinE1. AD, constructs in the pGAD-T7 vector backbone; BD, constructs in the pGBK-T7 vector backbone; AbA, 40 ng/ml of Aureobasidin A; Yeast cells grown in different types of medium. Two biological replicates were performed. **(B)** Pull-down analysis of the interactions of AtMinD1 or *cdm75* with ARC6, MCD1, and AtMinE1. Recombinant His-ARC6, His-MCD1, and His-AtMinE1 binds to GST-AtMinD1 or GST-AtMinD1(R49H). Glutathione Sepharose 4B beads were treated with a buffer only (lane 2) or coated with GST (lane 3), GST-AtMinD1 (lane 4), or GST-AtMinD1(R49H) (lane 5). The beads were then incubated with crude extracts of *Escherichia coli* cells expressing His-ARC6, His-MCD1, or His-AtMinE1. Proteins were eluted and analyzed by immunoblotting with anti-His and anti-GST antibodies.

Unexpectedly, the BiFC assay revealed a previously unknown interaction between AtMinD1_1__–__62_ and ARC6, and that the R49H mutation could reduce this interaction (Figure 4). Therefore, our research focused on verifying the interaction between ARC6 and AtMinD1. To detect the influence of point mutation on the interaction between the integrated AtMinD1 and these chloroplast division proteins, full-length AtMinD1, copied from the wild-type or *cdm75*, was used for the interaction analysis. First, a yeast two-hybrid assay was performed to investigate the interaction of AtMinD1 with ARC6, MCD1, and AtMinE1. As yeast cells co-transfected with full-length *AtMinD1* and *ARC6* or *MCD1* could grow well on a synthetic dropout (SD) medium lacking histidine with extra AbA (Figure 5A), this indicated that AtMinD1 could interact directly with MCD1 and ARC6. In contrast, it was difficult to grow the yeast cells co-introduced with AtMinD1(R49H) and ARC6 or MCD1 on this medium (Figure 5A), suggesting that the point mutation of residue 49 of AtMinD1 reduced the interaction between AtMinD1 and ARC6 or MCD1. Meanwhile, yeast cells co-transfected with *AtMinD1* and *AtMinE1* could grow well on the SD medium lacking histidine without AbA (Figure 5A). When considering our results with those of previous studies (Maple et al., 2005), we suggest that AtMinD1 can interact directly with AtMinE1, but that the interaction is extremely weak. The cells harboring AtMinD1(R49H) and AtMinE1 also could survive on the SD medium lacking histidine (Figure 5A), and this suggested that the point mutation of residue 49 of AtMinD1 may not affect the interaction between AtMinD1 and AtMinE1.

In addition, the interaction between AtMinD1 and the chloroplast division-related proteins (ARC6, MCD1, and AtMinE1) was further confirmed by *in vitro* pull-down assays. Recombinant His-ARC6 and His-MCD1 were precipitated notably from crude *E. coli* extracts by Glutathione-Sepharose beads coated with GST-tagged AtMinD1, but rarely by GST-AtMinD1(R49H)-coated beads and not by GST-coated beads (Figure 5B), which was consistent with the negative results from the yeast two-hybrid assays (Figure 5A). Unfortunately, recombinant His-AtMinE1 was not precipitated by the beads coated with GST-AtMinD1 or GST-AtMinD1(R49H) (Figure 5B). The above results indicated that AtMinD1 could interact directly with ARC6 and MCD1, and that the relationship between AtMinD1 and ARC6 or MCD1 was considerably weakened by the point mutation of residue 49 of AtMinD1, especially the interaction with ARC6. In addition, AtMinD1 may not interact with AtMinE1 directly or continuously, although the interaction may be extremely weak, as shown by the yeast two-hybrid assay.

### The Punctate Structure of AtMinD1 Is Suppressed in *arc6* Mutants

**FIGURE 6:**
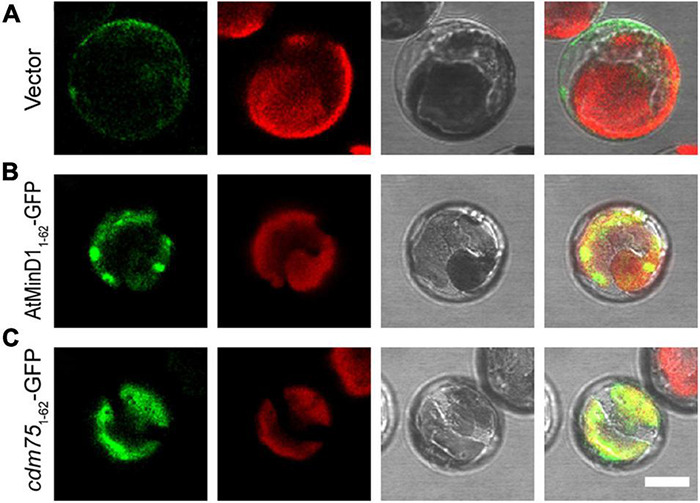
Localization of AtMinD1_1__–__62_ with GFP in *arc6* protoplasts. Fluorescence micrographs showing the N-terminal fragment of AtMinD1 fused with GFP transiently expressed in mutant *arc6* protoplasts, which include **(A)** GFP, **(B)** AtMinD1_1__–__62_-GFP, and **(C)** *cdm75*_1__–__62_-GFP. Scale bars = 10 μm.

The above data showed that point mutation of residue 49 of AtMinD1 could influence the AtMinD1-ARC6 interaction (Figure 5), and that the fluorescence of *cdm75*_1__–__62_-GFP was diffuse in the wild-type protoplasts (Figure 3). Therefore, based on our research, we assumed that the suppressed AtMinD1_1__–__62_-GFP–ARC6 interaction could directly influence the punctate structure of AtMinD1_1__–__62_-GFP. Significantly, the punctate localization of AtMinD1_1__–__62_-GFP was extremely dependent on the presence of ARC6. Therefore, AtMinD1_1__–__62_-GFP and *cdm75*_1__–__62_-GFP were transiently expressed in *arc6* mutant protoplasts, leading to detectable fluorescence. The results showed that the punctate localization of AtMinD1_1__–__62_-GFP could not be found: only several larger, bright aggregates of GFP fluorescence were detected in the giant chloroplasts of *arc6* plants (Figure 6). Thus, our research shows that the loss of function of ARC6 can partly influence the functional punctate phenotype of AtMinD1_1__–__62_. Furthermore, the fluorescence localization of *cdm75*_1__–__62_-GFP in *arc6* was similar to its position in the wild-type plants, with the fluorescence filling the entire chloroplast without obvious localization to spots (Figure 6). Thus, these data illustrate that the position of AtMinD1_1__–__62_ fragments may be partly affected by *arc6* in chloroplasts.

## Discussion

Based on previous research on *E. coli* and chloroplast division, the MinD protein, which has a significant role in the Min system, preventing the self-assembly of Z-rings everywhere but at the division site, was found initially in bacteria (de Boer et al., 1989; Miyagishima et al., 2005; Lutkenhaus, 2007; Rowlett and Margolin, 2013; Monahan et al., 2014). *MinD* genes have been identified in all available fully sequenced bacterial genomes and botanical genomes. Each of these organisms contained a single MinD-like gene (Colletti et al., 2000). Previous studies have described asymmetrical, single constriction sites during chloroplast division events in cells of *arc11, mind1-1*, and AtMinD1 antisense plants, regardless of whether the cells were mesophylls of mature leaves or hypocotyls and petioles of young seedlings (Marrison et al., 1999; Colletti et al., 2000; Fujiwara et al., 2004). In this study, we screened for and obtained an Arabidopsis mutant, *cdm75*, with large asymmetrically dividing chloroplasts, and was found to be induced by the R49H point mutation of AtMinD1 (Figure 1). Unlike other previously identified AtMinD1-related mutants, such as *arc11*, residue 49, which is the mutation site of *cdm75*, was located in the NCTS motif (residues 44–58) of AtMinD1, a highly conserved domain during the evolution of land plants (Figure 2 and Supplementary Figure 2).

The alignment of the MinD protein from various species shows that it is highly conserved in bacteria and plants (Fujiwara et al., 2004). Compared with other species, there is an additional conserved NCTS sequence of MinD in land plants (Figure 2 and Supplementary Figure 2). The phylogenetic tree also shows that MinD in plants has a single evolutionary origin (Supplementary Figure 2). Although the green plants are classified as a group, there are many differences between eukaryotic and prokaryotic plants (Figure 2). These data imply that the homolog of AtMinD1_44__–__58_ evolved from the common ancestor of land plants. Compared with algae, where there are only one or two chloroplasts in most cells, such as *Chlamydomonas reinhardtii* and *Cyanidioschyzon merolae*, whose chloroplast division is also strictly regulated by the cell cycle (de Vries and Gould, 2018), there are approximately 100 chloroplasts in higher terrestrial plant cells. Therefore, we speculated that the complicated Min system in land plants may be one of the effects of escaping from such a mono chloroplast bottleneck. Moreover, the R49H mutation in this conserved motif of AtMinD1 results in a prominent decrease in the number of chloroplasts in Arabidopsis (Figures 1A,B), and the asymmetrical division and large-chloroplast phenotype of *cdm75* resemble those of the bacterial Min phenotype (Figure 1C), rather than those of the division-inhibited filamentous phenotype (de Boer et al., 1989). Therefore, we presume that the emergence of the NCTS motif in ancestors of land plants is probably the key to the increased number of chloroplasts in higher terrestrial plants. However, the co-operative functions of other chloroplast fission proteins should not be ignored.

AtMinD1, a nuclear-encoded chloroplast protein (de Vries and Gould, 2018), has evolved a specific functional motif in the N-terminal, such as a transit peptide (TP), to allow transfer to the chloroplast (Lee and Hwang, 2018). A previous prediction by the ChloroP program (Emanuelsson et al., 1999) and other data (Fujiwara et al., 2004) showed that residues 1–62 of AtMinD1 comprised the transit peptide. Typically, chloroplast transit peptides do not display conservation at the primary structural level (Lee and Hwang, 2018), and their functionality is based on conserved physical properties, with lengths ranging from 20 to >100 residues (Bruce, 2000). However, the AtMinD1 highly conserved NCTS motif is from residues 44–58 (Figure 2). Therefore, we conclude that the length of the chloroplast transit peptide of AtMinD1 was shorter than 44 amino acids, which was consistent with the prediction of TargetP v2.0 (Supplementary Table 2). Meanwhile, our data revealed that the fused protein, AtMinD1_1__–__49_-GFP, could be successfully transited into chloroplast stroma, and that the mutation of residue 49 of AtMinD1 had no effect on the function of the TP (Figure 3). Thus, the above results support that the TP of AtMinD1 may be shorter than the initial 49 amino acids, or even occur before the NCTS domain (Figure 3). In wild-type chloroplasts, the punctate structure of AtMinD1_1__–__62_-GFP were dispersed on the chloroplast envelope (Figure 3), similar to the patterns reported in Nakanishi et al. (2009a) and Chen L. et al. (2018), and the punctate localization of AtMinD1_1__–__62_-GFP was prevented by *cdm75*_1__–__62_-GFP (Figure 3). From the above results, we concluded that the NCTS motif, which comprises the initial 62 amino acids except for the TP, played a significant role in the placement of the chloroplast division site, and that one feature of the N-terminal of AtMinD1, punctate localization, was probably determined by the NCTS domain.

In previous studies, many proteins related to chloroplast division have been found in the chloroplast stroma (Osteryoung and Pyke, 2014; Chen C. et al., 2018); of particular interest is the interaction between ARC6 and MCD1, which plays a major role in chloroplast division (Chen L. et al., 2018). Specifically, AtMinD1 can directly interact with MCD1 to prevent the self-assembly of the Z-ring everywhere but at the division site (Nakanishi et al., 2009a; Miyagishima et al., 2011). Similarly, the results revealed that the R49H mutation of AtMinD1 can reduce the interaction of AtMinD1_1__–__62_ with MCD1 *in vivo* (Figure 4). Previous studies (Maple et al., 2005, 2007) and our data have both shown that full-length AtMinD1 could also interact with MCD1 *in vitro*, and that the R49H mutation of AtMinD1 only has a small effect (Figure 5). According to the above data, we suggest that AtMinD1 has a domain, residues 63–349 of AtMinD1, which can significantly interact with MCD1 in yeast (Maple et al., 2005, 2007). Thus, this domain will weaken the influence of NTCS sequence on the interaction between full-length AtMinD1 and MCD1. However, the effect of the NCTS motif on this interaction cannot be ignored.

**FIGURE 7:**
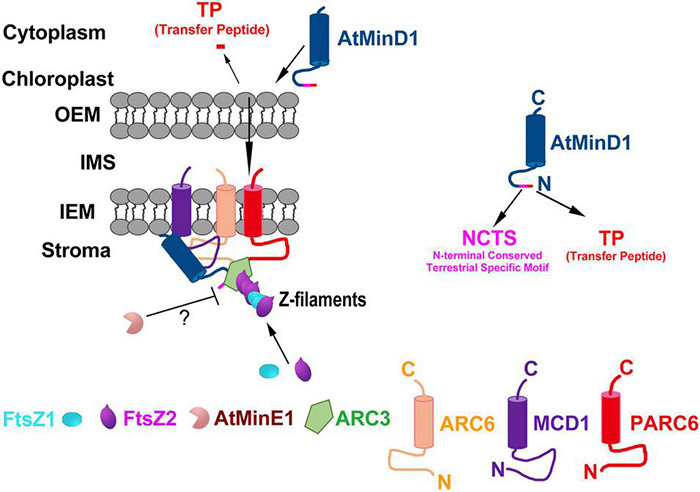
Proposed working model of the role of AtMinD1 in the onset of Z-ring positioning in chloroplasts. Model shows the topologies, interactions, and functional relationships of AtMinD1 with ARC6, FtsZ1, FtsZ2, MCD1, ARC3, AtMinE1, and PARC6 based on our genetic, cytological, and molecular data, emphasizing the role of AtMinD1 in the Min system. In this model, ARC6 interacts with FtsZ2 to tether heteropolymers (Z filaments) of FtsZ1 and FtsZ2 to the chloroplast inner envelope membrane (IEM), and PARC6 acts downstream of ARC6 to interact with FtsZ2 CTP to tether the Z filaments. The subsequent interaction between MCD1 and ARC6 through their stromal regions results in the recruitment of MCD1 to FtsZ heteropolymers coupled to ARC6 in the membrane. Coincidentally, AtMinD1 is enlisted and brought near chloroplasts, and then transferred to the stroma by cutting the transfer peptide. Subsequently, AtMinD1 is recruited by MCD1, and combines with ARC3 on the FtsZ heteropolymers and, in one of the most important steps of this process, AtMinD1 interacts with ARC6, permitting ARC3 to act in the disassembly of FtsZ filaments. In this process, the AtMinD1 NCTS motif plays a core role in the interaction, forming the chloroplast division complex and regulating the chloroplast division process. Finally, sufficient data exist in previous studies to affirm that AtMinE1 spatially restricts the activity of this inhibitory complex of MCD1, AtMinD1, and ARC3 *via* an unknown mechanism, although AtMinE1 might interact with AtMinD1 directly, resulting in Z-ring assembly. Many details of this model remain to be elucidated. The proteins shown do not represent stoichiometric ratios. OEM, outer envelope membrane; IMS, intermembrane space; N, N-terminus; C, C-terminus.

During chloroplast division, MCD1 is first enlisted to the Z-ring through the interaction with membrane-tethering protein ARC6 (Chen L. et al., 2018), and then AtMinD1 associates with the FtsZ filament protein *via* interaction with MCD1 to guide the chloroplast division (Nakanishi et al., 2009a). Hence, it is confirmed that ARC6 is upstream of AtMinD1. Interestingly, our data show that the punctate structure similar to the localization of AtMinD1_1__–__62_-GFP resulted from the interaction between ARC6 and AtMinD1_1__–__62_ (Figure 4). More specifically, several larger and bright aggregates of AtMinD1_1__–__62_-GFP, unlike wild-type protoplasts, appear in the *arc6* mutant protoplasts (Figure 6). Thus, we believe that ARC6 might be critical for the accumulation of AtMinD1 at division sites, and the function of AtMinD1 also depends on the presence of ARC6. Based on the above data, we hypnotized that AtMinD1 and ARC6 play a core function in Min complex for the determination of chloroplast division sites (Figure 7). In addition, we found that when Arg49 was changed to His in this sequence, the fragment could not condense into the clear aggregate in the *arc6* mutant (Figure 6). We assumed that the position of AtMinD1 could be influenced by interactions with unknown proteins, such as MCD1 or other chloroplast division-related proteins, in ARC6 loss of function situations, and that the R49H point mutation of AtMinD1 even impaired this interaction, obscuring the position of *cdm75*_1__–__62_-GFP.

A previous report has shown that AtMinD1 and ARC6 act in opposing directions: ARC6 promotes and AtMinD1 inhibits FtsZ filament formation in the chloroplast (Vitha et al., 2003). However, a direct interaction between ARC6 and AtMinD1 had not been found previously. In our research, we found that AtMinD1 could interact with ARC6 *in vitro* and *in vivo* (Figure 5). Further experiments showed the critical inner-stroma domain of ARC6, from amino acids 154 to 509, interacts directly with AtMinD1 (Supplementary Figure 4). Thus, there is now sufficient evidence to support the direct relationship between ARC6 and AtMinD1. Previous functional analyses of AtMinD1, especially the analysis of interaction relationships between AtMinD1 and other chloroplast division-related proteins, have always neglected the existence of the NCTS motif, and it is even regarded as a part of the TP that needs to be cleaved and remain in the cytoplasm. This may be the main reason that the AtMinD1-ARC6 interaction has not been found; notably, the interaction between AtMinD1_63__–__439_ and ARC6 could not be detected, even in our data (Supplementary Figure 5). However, we found that the AtMinD1-ARC6 interaction could be abolished by mutating residue 49 (Figure 5). Therefore, we assumed that after forming the chloroplast division-related protein complex, ARC6 and AtMinD1 play opposing roles in guiding chloroplast division (Vitha et al., 2003) *via* their interaction by the NCTS motif of AtMinD1.

Furthermore, our data confirmed that the interaction of AtMinD1 with AtMinE1 guides the placement of FtsZ filaments (Maple et al., 2005), but that this interaction is extremely weak (Figure 5A). Unexpectedly, given that AtMinD1 can interact with AtMinE1 *in vivo* (Figure 5A), but not *in vitro* (Figure 5B), we assume that their interaction may not be a permanent bond but occurs dynamically (Aldridge and Møller, 2005). Hence, we believe that AtMinD1 and AtMinE1 are nearby to each other in the chloroplast stroma or may have a slight interaction within the initial 62 amino acids of AtMinD1 (Figure 4). The influence of AtMinD1-AtMinE1 interaction caused by AtMinD1 NCTS motif needs further study.

According to the above data, AtMinD1 can interact with various chloroplast division-related proteins, such as ARC6 (Figure 5), MCD1 (Figure 5; Nakanishi et al., 2009a), AtMinE1 (Figure 5; Maple et al., 2005), and ARC3 (Maple et al., 2007), to form a complex to guide chloroplast division *via* their interaction (Supplementary Table 3). The above data demonstrate that the regulation of chloroplast division determination is more complicated than for prokaryotic ancestors. Specifically, in *Arabidopsis*, AtMinD1 is synthesized in the cytoplasm and transferred to the chloroplast stroma, guided by the TP. Subsequently, mature AtMinD1 participates in the chloroplast-division complex after it is recruited by MCD1, and then the stable AtMinD1-ARC6-MCD1 complex is formed by the close interaction of AtMinD1 with ARC6 at chloroplast division sites (Figure 7). Furthermore, the Min complex is formed with the participation of chloroplast division-related proteins (AtMinD1, ARC6, PARC6, ARC3, MCD1, and AtMinE1), and the Min complex regulates the chloroplast division site to ensure the division site is at the correct site of the mid chloroplast (Figure 7). However, the mutation of residue 49 in AtMinD1, located in the NCTS motif, can suppress or break the relationship between AtMinD1 and ARC6 or MCD1 (Figures 4, 5), so the chloroplast-division-related protein polymer cannot be condensed tightly, resulting in the inhibition of the chloroplast division process. This type of regulatory mechanism of chloroplast division, resulting from the special relationship between the N-terminal specific motif and chloroplast division-related proteins, is rarely found in chloroplast division-related research and provides a new avenue for the study on chloroplast division.

## Data Availability Statement

The datasets presented in this study can be found in online repositories. The names of the repository/repositories and accession number(s) can be found in the article/Supplementary Material.

## Author Contributions

YHu conceived the project. YHu, YZ, and XZ designed the experiments. YZ and XZ performed most of the experiments and analyzed the data. The other authors assisted in the experiments and discussed the results. XZ, YZ, and YHu wrote the manuscript.

## Conflict of Interest

The authors declare that the research was conducted in the absence of any commercial or financial relationships that could be construed as a potential conflict of interest.

## Publisher’s Note

All claims expressed in this article are solely those of the authors and do not necessarily represent those of their affiliated organizations, or those of the publisher, the editors and the reviewers. Any product that may be evaluated in this article, or claim that may be made by its manufacturer, is not guaranteed or endorsed by the publisher.

## Footnotes

1
http://www.crisoptical.com/lm2_41_344.htm

2
http://www.ncbi.nlm.nih.gov

3
http://www.jgi.doe.gov/JGI_microbial/html/index.html

4
http://www.kazusa.or.jp/cyano/

5
http://www.inra.fr/Internet/Produits/Predotar/

6
https://www.expasy.org/

7
http://pfam.xfam.org/

8
http://www.predictprotein.org/
