# Supplementary material for: Residue 49 of AtMinD1 Plays a Key Role in the Guidance of Chloroplast Division by Regulating the ARC6-AtMinD1 Interaction
Source: Front Plant Sci. 2021 Nov 22;12:752790. doi: 10.3389/fpls.2021.752790 (PMC8646090; doi:10.3389/fpls.2021.752790)
Supplement: Supplementary file 1 [file Data_Sheet_1.docx]

Supplementary Material

**Supplementary Table 1. Characteristics of genes encoding the MinD protein in different species.**

| **No.** | **Gene ID** | **Organism** | **NCBI BLAST name** |
| --- | --- | --- | --- |
| 1 | XP_002872087 | *Arabidopsis lyrata* | Eudicot |
| 2 | At5g24020 | *Arabidopsis thaliana* | Eudicot |
| 3 | XP_006289265 | *Capsella rubella* | Eudicot |
| 4 | ACG70180 | *Brassica oleracea var. botrytis* | Eudicot |
| 5 | XP_006394660 | *Eutrema salsugineum* | Eudicot |
| 6 | XP_006422026 | *Citrus clementina* | Eudicot |
| 7 | EPS62148 | *Genlisea aurea* | Eudicot |
| 8 | XP_004513184 | *Cicer arietinum* | Eudicot |
| 9 | XP_007152160 | *Phaseolus vulgaris* | Eudicot |
| 10 | KRH38382 | *Glycine max* | Eudicot |
| 11 | NP_001305534 | *Solanum tuberosum* | Eudicot |
| 12 | XP_010317508 | *Solanum lycopersicum* | Eudicot |
| 13 | NP_001313100 | *Nicotiana tabacum* | Eudicot |
| 14 | XP_011654648 | *Cucumis sativus* | Eudicot |
| 15 | XP_002273527 | *Vitis vinifera* | Eudicot |
| 16 | XP_007038802 | *Theobroma cacao* | Eudicot |
| 17 | XP_021290014 | *Herrania umbratica* | Eudicot |
| 18 | XP_007218343 | *Prunus persica* | Eudicot |
| 19 | XP_004308055 | *Fragaria vesca subsp. vesca* | Eudicot |
| 20 | XP_002305909 | *Populus trichocarpa* | Eudicot |
| 21 | AFC37490 | *Manihot esculenta* | Eudicot |
| 22 | XP_002513656 | *Ricinus communis* | Eudicot |
| 23 | Aqcoe7G025000.1 | *Aquilegia coerulea* | Flowering plant |
| 24 | CKAN_01546900 | *Cinnamomum kanehirae* | Flowering plant |
| 25 | Zosma76g00500.1 | *Zostera marina* | Monocot |
| 26 | Spipo11G0017000 | *Spirodela polyrhiza* | Monocot |
| 27 | XP_020573241.1 | *Phalaenopsis equestris* | Monocot |
| 28 | PKU68372.1 | *Dendrobium catenatum* | Monocot |
| 29 | PKA50129 | *Apostasia shenzhenica* | Monocot |
| 30 | XP_010941155.1 | *Elaeis guineensis* | Monocot |
| 31 | KAG1359324 | *Cocos nucifera* | Monocot |
| 32 | XP_017699551.1 | *Phoenix dactylifera* | Monocot |
| 33 | XP_009418620.1 | *Musa acuminata* | Monocot |
| **No.** | **Gene ID** | **Organism** | **NCBI BLAST name** |
| 34 | RWW21546.1 | *Ensete ventricosum* | Monocot |
| 35 | XP_039123399.1 | *Dioscorea cayenesisi* | Monocot |
| 36 | BAJ96739 | *Hordeum vulgare subsp. vulgare* | Monocot |
| 37 | XP_003557269 | *Brachypodium distachyon* | Monocot |
| 38 | XP_015642930 | *Oryza sativa Japonica Group* | Monocot |
| 39 | NP_001167965 | *Zea mays* | Monocot |
| 40 | XP_004964290 | *Setaria italica* | Monocot |
| 41 | XP_002436332 | *Sorghum bicolor* | Monocot |
| 42 | JAG88837 | *Wollemia nobilis* | gymnosperm |
| 43 | JAG94158 | *Araucaria cunninghamii* | gymnosperm |
| 44 | GBKT01018435 | *Ephedra trifurca* | gymnosperm |
| 45 | GBYR01021518 | *Ginkgo biloba* | gymnosperm |
| 46 | Azfi_s0355.g066723 | *Azolla filiculoides* | Fern |
| 47 | Sacu_v1.1_s0042.g012692 | *Salvinia cucullata* | Fern |
| 48 | XP_002961418 | *Selaginella moellendorffii* | Club-mosses |
| 49 | utg000045l | *Anthoceros puncatatus* | Hornwort |
| 50 | OAE27290 | *Marchantia polymorpha* | Livewort |
| 51 | Sphfalx0082s0019 | *Sphagnum palustre* | Moss |
| 52 | Sphfalx0128s0058 | *Sphagnum palustre* | Moss |
| 53 | KAG0626506.1 | *Ceratodon purpureus* | Moss |
| 54 | Pp3c1_24820V3.1 | *Physcomitrella patens* | Moss |
| 55 | Pp3c2_15820V3.2 | *Physcomitrella patens* | Moss |
| 56 | XP_006829073.1 | *Amborella trichopoda* | Flowering plant，the basal angiosperm |
| 57 | Nycol.D00867 | *Nymphaea colorata* | Flowering plant，the basal angiosperm |
| 58 | KFL00373_0040 | *Klebsormidium flaccidum* | Green algae |
| 59 | XP_003056998 | *Micromonas pusilla CCMP1545* | Green algae |
| 60 | XP_003075230 | *Ostreococcus tauri* | Green algae |
| 61 | XP_007514271 | *Bathycoccus prasinos* | Green algae |
| 62 | NP_038435 | *Mesostigma viride* | Green algae |
| 63 | YP_001019156 | *Chlorokybus atmophyticus* | Green algae |
| 64 | NP_050910 | *Nephroselmis olivacea* | Green algae |
| 65 | CAB53105 | *Prototheca wickerhamii* | Green algae |
| 66 | YP_636277 | *Pseudendoclonium akinetum* | Green algae |
| 67 | YP_005089924 | *Pedinomonas minor* | Green algae |
| 68 | YP_635891 | *Oltmannsiellopsis viridis* | Green algae |
| 69 | YP_004347767 | *Chlorella variabilis* | Green algae |
| 70 | AGZ19372 | *Chlorella sp. ArM0029B* | Green algae |
| 71 | NP_045875 | *Chlorella vulgaris* | Green algae |
| 72 | YP_003058345 | *Parachlorella kessleri* | Green algae |
| **No.** | **Gene ID** | **Organism** | **NCBI BLAST name** |
| 73 | ACQ90760 | *Oocystis solitaria* | Green algae |
| 74 | XP_001697031 | *Chlamydomonas reinhardtii* | Green algae |
| 75 | XP_002947961 | *Volvox carteri f. nagariensis* | Green algae |
| 76 | YP_001382148 | *Leptosira terrestris* | Green algae |
| 77 | ABX82607 | *Trebouxia aggregata* | Green algae |
| 78 | YP_004221983 | *Coccomyxa subellipsoidea C-169* | Green algae |
| 79 | YP_006666448 | *Trebouxiophyceae sp. MX-AZ01* | Green algae |
| 80 | WP_015168058 | *Synechococcus sp. PCC 7502* | Cyanobacteria |
| 81 | WP_015125087 | *Synechococcus sp. PCC 6312* | Cyanobacteria |
| 82 | WP_015191143 | *Gloeocapsa sp. PCC 7428* | Cyanobacteria |
| 83 | WP_015079597 | *Anabaena sp. 90* | Cyanobacteria |
| 84 | WP_000101055 | *Escherichia coli* | Bacteria |

**Supplementary Table 2. Transfer peptide prediction of AtMinD1 subcellular localization.**

| Subcellular localization of TargetP (plant) V2.0 | | | | | |
| --- | --- | --- | --- | --- | --- |
| Protein type | Chloroplast transfer peptide | Mitochondrial transfer peptide | Thylakoid luminal transfer peptide | Signal peptide | Other |
| Likelihood | 0.6098 | 0.3657 | 0.004 | 0.0001 | 0.0204 |

Prediction result: The cleavage site position 43 and 44: IRS-VL. Probability: 0.2492.

**Supplementary Table 3. The interactions between different chloroplast-division-related proteins.**

|  | ARC6 | PARC6 | AtMinD1 | AtMinE1 | FtsZ1 | FtsZ2 | ARC3 | MCD1 |
| --- | --- | --- | --- | --- | --- | --- | --- | --- |
| ARC6 | × | ？ | ？ | ？ | ？ | ？ | × | √ |
| PARC6 | × | ？ | ？ | ？ | ？ | √ | √ | × |
| AtMinD1 | ○ | ？ | √ | √ | ？ | ？ | √ | √ |
| AtMinE1 | ？ | ？ | √ | √ | ？ | ？ | √ | ? |
| FtsZ1 | × | ？ | ？ | ？ | √ | √ | √ | × |
| FtsZ2 | √ | √ | ？ | ？ | √ | √ | √ | × |
| ARC3 | × | √ | √ | √ | √ | √ | ？ | ？ |
| MCD1 | √ | × | √ | ? | × | × | ？ | ？ |

○: Detected in our study; √: Identified; ×: Not found; ?: Undetected

**Supplementary Table 4. Primers used in this study.**

| **primer name** | **Sequence (5’-3’)** | **Function** |
| --- | --- | --- |
| 5-85L | GGAACATATGTGCTGAGATAAT | The primer of mapping *cdm75* locus on the chromosome V. |
| 5-85R | TTTAAAAATAACTGAACGTCCT | The primer of mapping *cdm75* locus on the chromosome V. |
| nga249L | GGATCCCTAACTGTAAAATCCC | The primer of mapping *cdm75* locus on the chromosome V. |
| nga249R | TACCGTCAATTTCATCGCC | The primer of mapping *cdm75* locus on the chromosome V. |
| SO262L | ATCATCTGCCCATGGTTTTT | The primer of mapping *cdm75* locus on the chromosome V. |
| SO262R | TTGCTTTTTGGTTATATTCGGA | The primer of mapping *cdm75* locus on the chromosome V. |
| 5-58L | TAACGCTCTTTGGCGATCTT | The primer of mapping *cdm75* locus on the chromosome V. |
| 5-58R | ATTGAGTCCGGTTGAGCATC | The primer of mapping *cdm75* locus on the chromosome V. |
| W-001 | CATGCCATGGCTATGGCGTCTCTGAGATTGTTCTC | Cloning pCambia1300-35S-AtMinD1 |
| W-002 | GGTAACCTTAGCCGCCAAAGAAAGAGAAGAAGCC | Cloning pCambia1300-35S-AtMinD1 |
| HY-003 | GACTAGTGGTGATAACGACGATACGCG | Cloning pCambia1300-35S-AtMinD1_1-64_-GFP |
| M-011 | CGGAATTCGCGATTAAATTGAAGAACGGATC | Cloning pSAT1-AtMinD1_1-49_-GFP |
| M-012 | CGGAATTCGTGATTAAATTGAAGAACGGATC | Cloning pSAT1-AtMinD1_1-49_(R49H)-GFP |
| M-015 | CGGAATTCGACGATACGCGGCGTTTCTCC | Cloning pSAT1-AtMinD1_1-61_-GFP and pSAT1-AtMinD1_1-61_(R49H)-GFP |
| M-016 | CGGGATCCAACGACGATACGCGGCGTTTC | Cloning pSAT1-AtMinD1_1-62_-GFP and Cloning pSAT1-AtMinD1_1-62_(R49H)-GFP |
| M-017 | CGGGATCCGGTGATAACGACGATACGCGG | Cloning pSAT1-AtMinD1_1-64_-GFP and Cloning pSAT1-AtMinD1_1-64_(R49H)-GFP |
| HY-053 | CATTACTCCGGCGAATGAAG | RT-PCRs for total *AtMinD1* transcript levels |
| HY-054 | CGTGCTTCGAATAACCTCAG | RT-PCRs for total *AtMinD1* transcript levels |
| HY-055 | TCCTAGGACAATGTGGAATTCTACTG | RT-PCRs for endogenous *AtMinD1* transcript levels |
| HY-056 | CAGAAATCAAGAACCTCAAGAACAAA | RT-PCRs for endogenous *AtMinD1* transcript levels |
| HY-045 | GTCGTACAACCGGTATTGTG | RT-PCRs for actinⅡ |
| HY-046 | GAGCTGGTCTTTGAGGTTTC | RT-PCRs for actinⅡ |
| HY-075 | TACATATGGCGTCTCTGAGATTGTTC | Cloning pGBKT7-AtMinD1 and pGADT7-AtMinD1 |
| HY-076 | ATGGATCCTTAGCCGCCAAAGAAAGAGAAGAAGCC | Cloning pGBKT7-AtMinD1 and pGADT7-AtMinD1 |
| Z-001 | TACATATGGAAGCTCTGAGTCACGTCG | Cloning pGADT7-ARC6 |
| Z-002 | ATCCCGGGTTATGATGCAAGAACAGAGCCTTC | Cloning pGADT7-ARC6 |
| Z-003 | TACATATGGCATCCATTGATTCTCTC | Cloning pGBKT7-MCD1 |
| Z-004 | ATCCCGGGTCAGGACTTGTGCGTTTCCT | Cloning pGBKT7-MCD1 |
| Z-005 | ATCATATGGCGATGTCTTCTGGAAC | Cloning pGBKT7-AtMinE1 and p-ET28a (+)-AtMinE1 |
| Z-006 | ATGGATCCTCACTCTGGAACATAAAAATCG | Cloning pGBKT7- AtMinE1 and p-ET28a (+)-AtMinE1 |
| Z-007 | CGGGATCCATGGCGTCTCTGAGATTGTT | Cloning pGEX-KG-AtMinD1 |
| Z-008 | CGGAATTCGCCGCCAAAGAAAGAG | Cloning pGEX-KG-AtMinD1 |
| Z-009 | GCGTCGACTTATGATGCAAGAACAGAGCCTTC | Cloning p-ET28a (+)-ARC6 |
| Z-010 | GCGTCGACTCAGGACTTGTGCGTTTCCT | Cloning p-ET28a (+)-MCD1 |
| M-021 | CATGCCATGGCTATGGCGTCTCTGAGATTGTTCTC | Cloning pSAT1-cGFP^C^–AtMinD1_1-62_ and pSAT1-cGFP^C^–cdm75_1-62_ |
| M-022 | GCGTCGACCAACGACGATACGCGGCGTTT | Cloning pSAT1-cGFP^C^–AtMinD1_1-62_ and pSAT1-cGFP^C^–cdm75_1-62_ |
| M-024 | CATGCCATGGCTATGGAAGCTCTGAGTCACGTCG | Cloning pSAT1-cGFP^N^-ARC6 |
| M-025 | GCGTCGACCTGATGCAAGAACAGAGCCTTCA | Cloning pSAT1-cGFP^N^-ARC6 |
| M-026 | CATGCCATGGCTATGGCATCCATTGATTCTCTCC | Cloning pSAT1-cGFP^N^-MCD1 |
| M-027 | GCGTCGACCGGACTTGTGCGTTTCCTCAGA | Cloning pSAT1-cGFP^N^-MCD1 |
| M-028 | CATGCCATGGCTATGGCGATGTCTTCTGGAACTCT | Cloning pSAT1-cGFP^N^-AtMinE1 |
| M-029 | GCGTCGACCCTCTGGAACATAAAAATCGAACC | Cloning pSAT1-cGFP^N^-AtMinE1 |


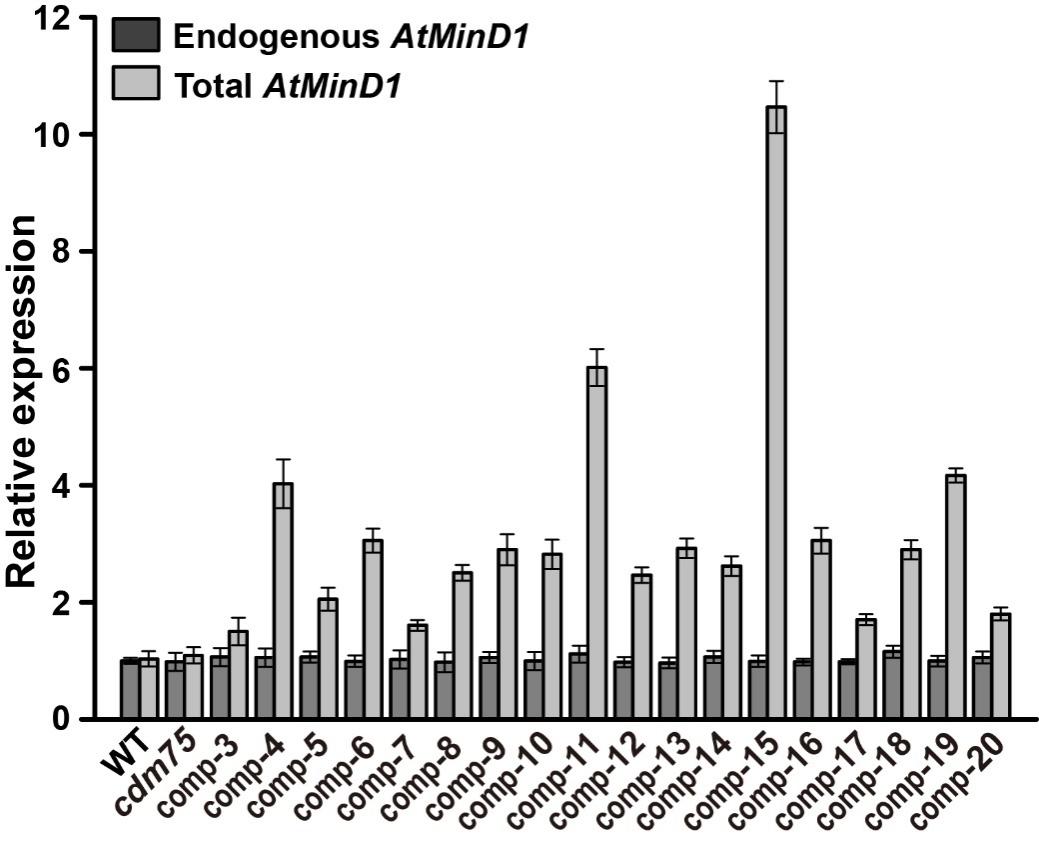


**Supplementary Figure 1.** Quantification of endogenous and total AtMinD1 transcripts in Arabidopsis WT, *cdm75* and 18 incomplement 35S-AtMinD1-HA/*cdm75* transgenic lines (*comp-3*, *comp-4*, *comp-5*, *comp-6*, *comp-7*, *comp-8*, *comp-9*, *comp-10*, *comp-11, comp-12*, *comp-13, comp-14, comp-15, comp-16, comp-17, comp-18, comp-19,* and *comp-20*).


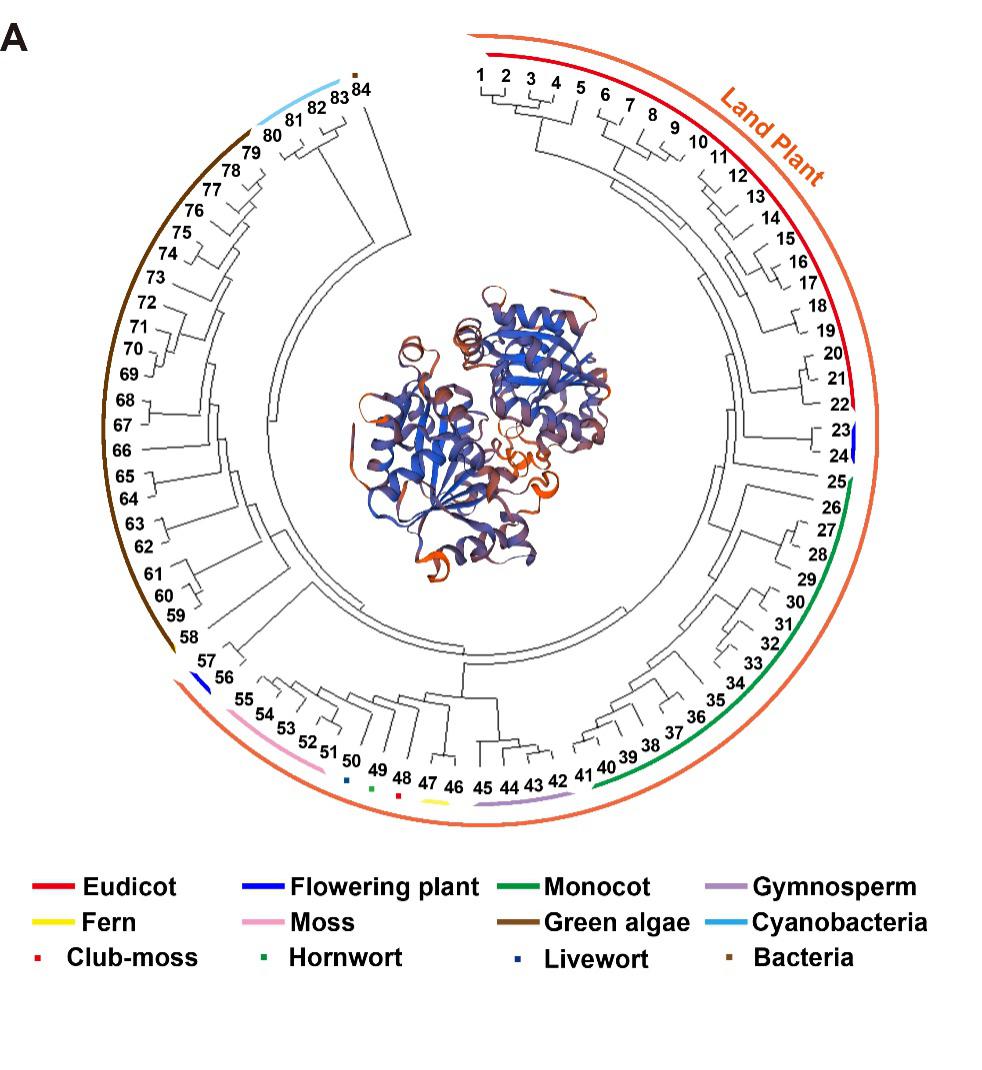


**Supplementary Figure 2.** **Unrooted dendrogram of MinD proteins from different species.** Sequence alignments were performed using the ClustalW algorithm and an unrooted dendrogram was drawn. DNA and protein sequence databases were obtained from the National Center for Biotechnology Information database (<http://www.ncbi.nlm.nih.gov>). Preliminary sequence data for most cyanobacterial genomes were obtained from the Department of Energy Joint Genome Institute (http:// [www.jgi.doe.gov/JGI_microbial/html/index.html](http://www.jgi.doe.gov/JGI_microbial/html/index.html)) and from the Kazusa DNA Research Institute of Japan (<http://www.kazusa.or.jp/cyano/>). The *Chlamydomonas reinhardtii* genomic sequence was obtained from <http://www.biology.duke.edu/chlamy_genome/blast/blast_form.html>. Protein and DNA similarity searches were performed using Basic Local Alignment Search Tool (TBLASTN and BLASTN; Altschul et al., 1990). The MinD proteins in different species are indicated by different colors, and AtMinD1, which is the homolog of the MinD protein in Arabidopsis, was classified in Eudicot, signed No. 2. The 3D protein structure is the dimer of the AtMinD1 foreseen by Swissmodle (https://swissmodel.expasy.org/interactive/6JQ5Q5/models/). The number corresponds to the genes encoding MinD protein in different species in Table S1.


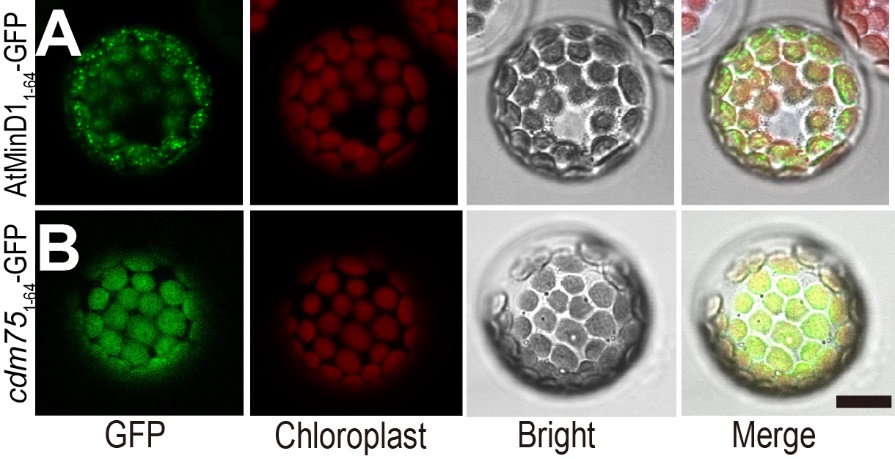


**Supplementary Figure 3.** **Fluorescence micrograph assays of AtMinD1_1–64_ with AtMinD1 or *cdm75* fused with GFP in Arabidopsis protoplasts.**

**
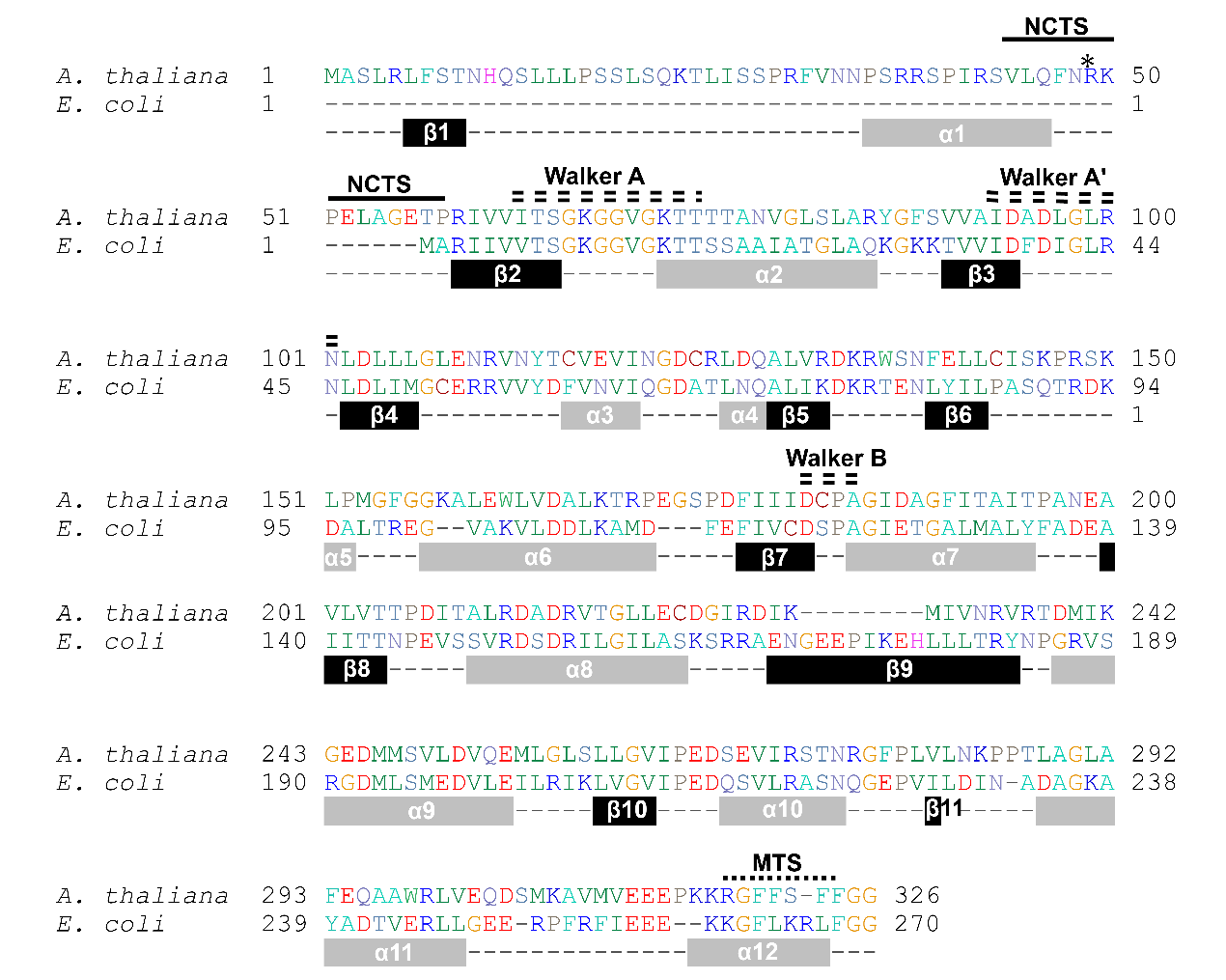
**

**Supplementary Figure 4. Sequence alignment of MinD proteins.** Sequence alignment of MinD proteins from *Arabidopsis thaliana* (database accession number AB030278), *Escherichia coli* (J03153) with secondary structure elements based on structural (Hayashi et al., 2001; Sakai et al., 2001) and membrane localization (Szeto et al., 2002; Zhou and Lutkenhaus, 2003) analysis, and a secondary structure prediction program (Rost et al. 2003). A single base substitution of AtMinD1 at position 49, changing Arg(CGG) to His(CAC), is indicated by the asterisk.


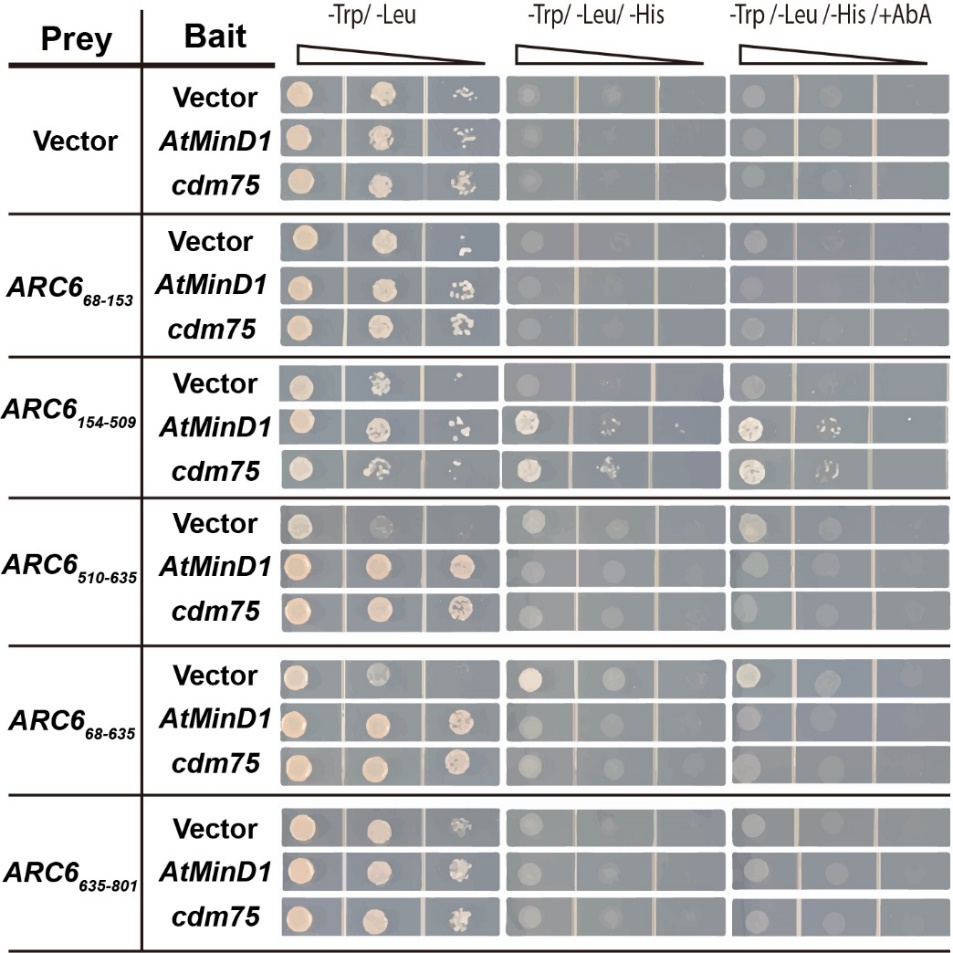


**Supplementary Figure 5.** **The interaction between AtMinD1 or *cdm75* and ARC6_154–509_.**


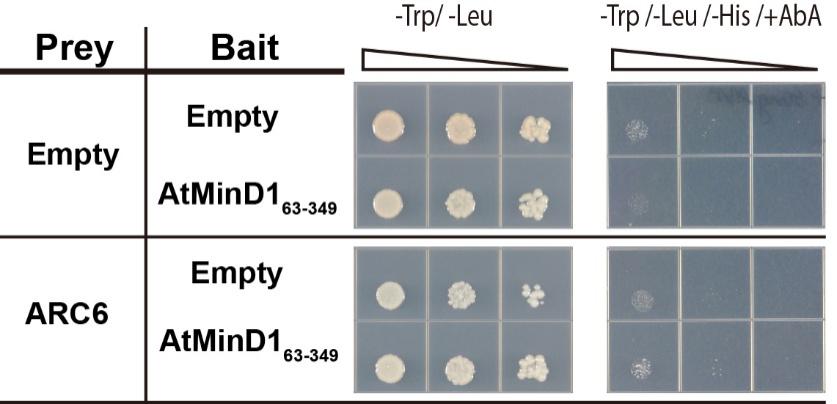


**Supplementary Figure 6. The interaction between AtMinD1_63-349_ and ARC6 by yeast two-hybrid assay.**


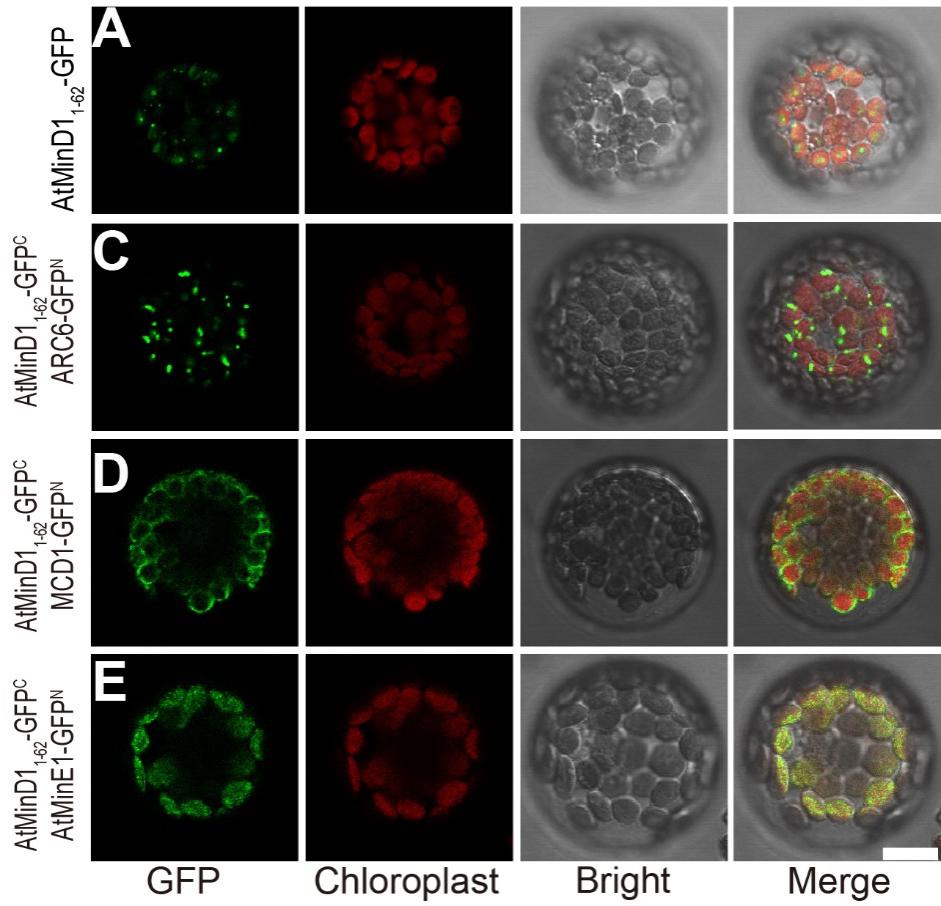


**Supplementary Figure 7. Clearer and enlarged images, from a high-power microscope, showing the position of AtMinD1_1-62_ and the interaction between AtMinD1_1-62_ and chloroplast division-related proteins (ARC6, MCD1, and AtMinE1) by BiFC assay in Arabidopsis protoplasts.**

**Supplementary Figure 8. The analysis of the relative quantification of His-MCD1 in the pull-down assay in Figure 5B.**
